# Chronic Lymphocytic Leukemia: 2025 Update on the Epidemiology, Pathogenesis, Diagnosis, and Therapy

**DOI:** 10.1002/ajh.27546

**Published:** 2025-01-28

**Authors:** Michael Hallek

**Affiliations:** ^1^ Department I of Internal Medicine and Medical Faculty University of Cologne Köln Germany; ^2^ Center for Integrated Oncology Aachen Bonn Köln Düsseldorf Köln Germany; ^3^ Center of Excellence on “Cellular Stress Responses in Aging‐Associated Diseases,” University of Cologne Köln Germany; ^4^ Center of Cancer Research Cologne Essen Köln Germany; ^5^ National Center for Tumor Diseases (NCT) West Köln Germany

**Keywords:** chronic lymphocytic leukemia, diagnostic work up, epidemiology, management and therapy, molecular and cellular pathogenesis

## Abstract

**Disease Overview:**

Chronic lymphocytic leukemia (CLL) is the most frequent type of leukemia. It typically occurs in older patients and has a highly variable clinical course. Leukemic transformation is initiated by specific genomic alterations that interfere with the regulation of proliferation and apoptosis in clonal B‐cells.

**Diagnosis:**

The diagnosis is established by blood counts, blood smears, and immunophenotyping of circulating B‐lymphocytes, which identify a clonal B‐cell population carrying the CD5 antigen as well as typical B‐cell markers.

**Prognosis and Staging:**

Two clinical staging systems, Rai and Binet, provide prognostic information by using the results of physical examination and blood counts. Various biological and genetic markers provide additional prognostic information. Deletions of the short arm of chromosome 17 (del(17p)) and/or mutations of the *TP53* gene predict a shorter time to progression with most targeted therapies. The CLL international prognostic index (CLL‐IPI) integrates genetic, biological, and clinical variables to identify distinct risk groups of patients with CLL. The CLL‐IPI retains its significance in the era of targeted agents, but the overall prognosis of CLL patients with high‐risk stages has improved.

**Therapy:**

Only patients with active or symptomatic disease or with advanced Binet or Rai stages require therapy. When treatment is indicated, several therapeutic options exist: combinations of the BCL2 inhibitor venetoclax with obinutuzumab, or venetoclax with ibrutinib, or monotherapy with one of the inhibitors of Bruton tyrosine kinase (BTK). At relapse, the initial treatment may be repeated if the treatment‐free interval exceeds 3 years. If the leukemia relapses earlier, therapy should be changed using an alternative regimen.

**Future Challenges:**

Combinations of targeted agents now provide efficient therapies with a fixed duration that generate deep and durable remissions. These fixed‐duration therapies have gained territory in the management of CLL, as they are cost‐effective, avoid the emergence of resistance, and offer treatment free time to the patient. The cure rate of these novel combination regimens is unknown. Moreover, the optimal sequencing of targeted therapies remains to be determined. A medical challenge is to treat patients who are double‐refractory to both BTK and BCL2 inhibitors. These patients need to be treated within experimental protocols using novel drugs.

## 1 Introduction and Disease Overview

In the most recent update of the SEER database, the age‐adjusted incidence of chronic lymphocytic leukemia (CLL) was 4.6 per 100 000 inhabitants per year [1], which makes CLL the most common type of leukemia. The median age at diagnosis is 70 years [1]. Less than 10% of patients with CLL are younger than 45 years. More male than female patients (1.9:1) are affected, and this gender effect seems to be stable across all ethnicities.

Approximately 0.6% of men and women will be diagnosed with CLL at some point during their lifetime. For 2024, SEER estimates 20 700 new CLL cases in the US, which represents 1% of all new cancer cases. In 2024, an estimated 215 107 people were living with chronic lymphocytic leukemia in the United States [1]. While the incidence of CLL has been stable over the last two decades, the mortality is continuously declining. CLL is estimated to cause 4440 deaths in 2024, representing 0.7% of all cancer deaths. The CLL‐related death rate was 1.1 per 100 000 men and women per year. The 5‐year relative survival of patients with CLL was 65.1% in 1975 and has steadily increased over the past decades; it is estimated at 88.5% in 2024 [1]. Similar data regarding the epidemiology of CLL have been reported in Europe [2], while the incidence is lower in Asian countries and ethnicities [3, 4].

CLL is characterized by the clonal proliferation and accumulation of mature, typically CD5‐positive B‐cells within the blood, bone marrow, lymph nodes, and spleen [5]. The capacity to generate clonal B cells seems to be acquired at the hematopoietic stem cell (HSC) stage [6], suggesting that the primary leukemogenic event in CLL might involve multipotent, self‐renewing HSCs. The process of a stepwise leukemogenic transformation is increasingly understood. CLL is often initiated by the loss or addition of large chromosomal material (e.g., deletion 13q, deletion 11q, trisomy 12) followed later by additional mutations that render the leukemia increasingly aggressive [7].

Approximately 80% of all patients with CLL carry at least one of four **common chromosomal alterations**: a deletion in chromosome 13q14.3 (del(13q)), del(11q), del(17p), or trisomy 12 [8]. Del(13q) is the most common chromosomal alteration, occurring in about 55% of all cases. An isolated del(13q14) usually indicates a less aggressive form of the disease. The miRNAs miR‐15a and 16–1, located in the critical region of *del(13q14)* [9], regulate the expression of proteins that inhibit apoptosis or drive cell cycle progression [10]. Deletions of the short arm of chromosome 17 (del(17p)) are found in 5%–8% of chemotherapy‐naïve patients. These deletions almost always include band 17p13, where the tumor suppressor gene *TP53* is located. Patients with CLL carrying a del(17p) clone show marked resistance against genotoxic chemotherapies [11]. Mutations of *TP53* are found in 4%–37% of patients with CLL and are associated with inferior prognosis [12]. Among cases with confirmed del(17p), the majority show mutations in the remaining *TP53* allele (> 80%). In cases without del(17p), *TP53* mutations are much rarer but have a similarly detrimental effect on chemotherapy response and overall survival [13]. Deletions of the long arm of chromosome 11 (del(11q)) can be found in approx. 25% of chemotherapy‐naïve patients with advanced disease stages and 10% of patients with early‐stage disease [14, 15]. These deletions frequently encompass band *11q23* harboring the gene *ATM*, which encodes for the proximal DNA damage response kinase ATM. In addition, patients carrying a del(11q) clone typically show bulky lymphadenopathy, rapid progression, and reduced OS [16]. Interestingly, some of the poor prognostic features of del(11q) were overcome by chemoimmunotherapy [11]. Trisomy 12 is observed in 10%–20% of patients with CLL and is associated with an intermediate prognosis [8]. The genes involved in the pathogenesis of CLL carrying a trisomy 12 are largely unknown.

The use of whole exome sequencing has allowed us to characterize the **genomic landscape** of CLL. In addition to the above‐described chromosomal aberrations, a total number of 44 recurrently mutated genes and 11 recurrent somatic copy number variations have been found [7]. These include the genes *NOTCH1*, *MYD88*, *TP53*, *ATM*, *SF3B1*, *FBXW7*, *POT1*, *CHD2*, *RPS15*, *IKZF3*, *ZNF292*, *ZMYM3*, *ARID1A*, and *PTPN11* [7, 15, 17, 18]. These analyses identified RNA processing and export, MYC activity, and MAPK signaling as central pathways involved in CLL [7]. In addition, proteins involved in DNA damage signaling and DNA repair are frequently implicated [19]. Interestingly, both del(17p) and del(11q), as well as inactivating somatic mutations in *TP53* and *ATM*, are enriched in patients with secondary resistance to DNA‐damaging chemotherapy [15, 17]. Mutations in an enhancer located on chromosome 9p13 can reduce the expression of the B‐cell‐specific transcription factor PAX5 [18]. Robbe et al. have confirmed the relevance of genomic alterations including structural variants, copy number changes, and global genome features including telomere length, mutational signatures, and genomic complexity for clinical outcome [20].

The CLL **epigenome** has emerged as an additional disease‐defining feature [21, 22]. Expanding populations of CLL cells diversify by stochastic changes in DNA methylation called epimutations [23]. Multiplexed single‐cell reduced representation bisulfite sequencing of B‐cells from healthy donors and patients with CLL has provided new insights into changes in DNA methylation known as epimutations [24, 25]. The results suggest that the integration of genetic, epigenetic, and transcriptional information gained at a single cell level allows one to chart the lineage history of individual cases of CLL and their evolution with therapy.

More recently, attempts have been made to integrate several layers of biological and clinical variables into **comprehensive models**. Knisbacher et al. integrated genomic, transcriptomic, and epigenomic data from 1148 patients [26]. They identified 202 candidate genetic drivers of CLL (of which 109 were so far undiscovered) and refined the characterization of IGHV subtypes. The analyses identified new gene expression subtypes, which seemed to allow the subcategorization of CLL and to create independent prognostic categories.

Survival of CLL cells depends on a permissive **microenvironment** composed of cellular components like macrophages, fibroblasts, T cells, or stromal follicular dendritic cells [27, 28, 29, 30], providing stimuli for the activation of crucial survival and pro‐proliferative signaling pathways in transformed cells. This microenvironment produces various essential proteins (chemokines, cytokines, and angiogenic factors) that interact with leukemic cells via appropriate surface receptors or adhesion molecules to support the survival of CLL cells [29, 30, 31, 32]. Interestingly, some of the new inhibitors also exert their effects by targeting key pathways of microenvironmental cells in patients with CLL [33, 34, 35, 36, 37, 38]. The use of drugs that modulate the microenvironment may represent a new therapeutic strategy for relapsed or refractory CLL [39].

As a consequence of these advances in our understanding of the pathogenesis, the management of CLL continues to undergo highly relevant improvements. Several new drugs have been approved during the last three decades. Chemoimmunotherapies that combined fludarabine, cyclophosphamide with rituximab, or chlorambucil with obinutuzumab have improved overall survival when used as first‐line therapy for patients with CLL. More recently, specific inhibitors interrupting important pathways for CLL cell survival (Bruton tyrosine kinase, PI3 kinase, and BCL2) have been approved. These inhibitors have now replaced chemoimmunotherapy in first and second‐line indications. This updated review integrates the latest innovations in CLL therapy as well as diagnostic tools and provides an updated algorithm to guide diagnostic and therapeutic decisions in daily practice.

## 2 Diagnosis

The iwCLL guidelines [5] give clear recommendations on how to establish the diagnosis of CLL. In most cases, the diagnosis of CLL is established by blood counts, differential counts, a blood smear, and immunophenotyping. The 5th edition of the World Health Organization Classification of Hematolymphoid Tumors categorizes CLL into the group of mature B‐cell neoplasms. Within this category, CLL is placed in the category “pre‐neoplastic and neoplastic small lymphocytic proliferations category: MBL and CLL” [40]. This family comprises monoclonal B‐cell lymphocytosis (MBL) and CLL/SLL. CLL is described as leukemic lymphocytic lymphoma, distinguished from SLL by its leukemic appearance [40]. CLL is always a disease of neoplastic B‐cells, while the entity formerly described as T‐CLL is called T‐cell prolymphocytic leukemia (T‐PLL) [40, 41]. B‐prolymphocytic leukemia is no longer recognized as an entity.

**The diagnosis of CLL** requires the presence of ≥ 5000 B‐lymphocytes/μL in the peripheral blood for at least 3 months. The clonality of the circulating B‐lymphocytes needs to be confirmed by flow cytometry. The leukemia cells found in the blood smear are characteristically small, mature lymphocytes with a narrow border of cytoplasm and a dense nucleus lacking discernible nucleoli and having partially aggregated chromatin. These cells may be found admixed with larger or atypical cells, cleaved cells, or prolymphocytes, which may comprise up to 55% of the blood lymphocytes [42]. Finding prolymphocytes in excess of this percentage would favor a diagnosis of prolymphocytic leukemia (B‐cell PLL). Gumprecht nuclear shadows, or smudge cells, found as cell debris, are other characteristic morphologic features found in CLL.

**Monoclonal B lymphocytosis** [5]. In the absence of lymphadenopathy or organomegaly (as defined by physical examination or CT scans), cytopenias, or disease‐related symptoms, the presence of fewer than 5000 B‐lymphocytes per μL blood is defined as “monoclonal B‐lymphocytosis” (MBL) [43]. The presence of cytopenia caused by a typical marrow infiltrate defines the diagnosis of CLL regardless of the number of peripheral blood B‐lymphocytes or lymph node involvement. MBL seems to progress to frank CLL at a rate of 1%–2% per year [43].

The definition of SLL requires the presence of lymphadenopathy and the absence of cytopenias caused by a clonal marrow infiltrate. Moreover, the number of B‐lymphocytes in the peripheral blood should not exceed 5000/μL. In SLL, the diagnosis should be confirmed by histopathological evaluation of a lymph node biopsy whenever possible.

**Immunophenotyping** [5]. CLL cells co‐express the surface antigen CD5 together with the B‐cell antigens CD19, CD20, and CD23. The levels of surface immunoglobulin, CD20, and CD79b are characteristically low compared to those found in normal B cells [44, 45, 46]. Each clone of leukemia cells is restricted to the expression of either kappa or lambda immunoglobulin light chains [44]. The expression of CD5 can also be observed in other lymphoid malignancies, such as mantle cell lymphoma [47]. A recent, large harmonization effort has confirmed that a panel of CD19, CD5, CD20, CD23, kappa, and lambda is usually sufficient to establish the diagnosis [48]. In borderline cases, markers such as CD43, CD79b, CD81, CD200, CD10, or ROR1 may help to refine the diagnosis [48].

## 3 Risk Stratification, Staging, and Indication for Treatment

Two widely accepted clinical staging systems co‐exist [49, 50]. The Rai classification was later modified to reduce the number of prognostic groups from five to three [51]. Both systems describe three major prognostic groups with discrete clinical outcomes. These two staging systems are simple, inexpensive, and rely on a physical examination and standard laboratory tests. They do not require ultrasound, computed tomography, or magnetic resonance imaging.

The **Rai staging system** defines low‐risk disease as patients who have lymphocytosis with leukemia cells in the blood and/or marrow (lymphoid cells > 30%) (former Rai Stage 0). Patients with lymphocytosis, enlarged nodes in any site, and splenomegaly and/or hepatomegaly (lymph nodes being palpable or not) are defined as having intermediate‐risk disease (formerly considered Rai stage I or Stage II). High‐risk disease includes patients with disease‐related anemia (as defined by a hemoglobin (Hb) level less than 11 g/dL) (formerly Stage III) or thrombocytopenia (as defined by a platelet count of less than 100 × 10^9^/L) (formerly Stage IV).

The **Binet staging system** is based on the number of involved areas, as defined by the presence of enlarged lymph nodes greater than 1 cm in diameter or organomegaly, and on whether there is anemia or thrombocytopenia. The areas of involvement considered are (1) head and neck, including the Waldeyer ring (this counts as one area, even if more than one group of nodes is enlarged). (2) axillae (involvement of both axillae counts as one area). (3) Groins, including superficial femoral (involvement of both groins counts as one area). (4) Palpable spleen. (5) Palpable liver (clinically enlarged). The Binet staging system defines Stage A as Hb ≥ 10 g/dL and platelets ≥ 100 × 10^9^/L and up to two of the above involved; Stage B as Hb ≥ 10 g/dL and platelets ≥ 100 × 10^9^/L and organomegaly greater than that defined for Stage A (i.e., three or more areas of nodal or organ enlargement); and Stage C as Hb of less than 10 g/dL and/or a platelet count of less than 100 × 10^9^/L.

Due to recent progress in CLL therapy, the two clinical staging systems have become insufficient to distinguish prognostic subgroups [52]. A plethora of potential markers can provide prognostic information independent of the clinical stage [53]. in particular, some of the above‐described genetic and chromosomal aberrations. To condense the prognostic information to a few clinically relevant parameters, comprehensive scores have been constructed that combine clinical, biological, and genetic information [52, 54, 55, 56]. One of the most widely used prognostic scores is the **CLL International Prognostic Index** (**CLL‐IPI**) [57]. It uses a weighted grading of five independent prognostic factors: *TP53* deletion and/or mutation (collectively called *TP53* dysfunction), *IGHV* mutational status, serum β_2_‐microglobulin, clinical stage, and age. The CLL‐IPI separates four groups with different survival at 5 years (see Table 1). As the CLL‐IPI was created in the era of chemoimmunotherapy using trial data from different countries, its value was re‐analyzed recently in the era of targeted agents [58]. The CLL‐IPI maintains its prognostic value in predicting PFS outcomes with targeted drugs, but its impact in predicting survival appeared diminished. With a median observation time of 40.5 months, the 3‐year progression‐free survival (PFS) rates for targeted drug‐treated patients varied by CLL‐IPI risk group: 96.5% (low), 87.6% (intermediate), 82.4% (high), and 78.7% (very high). CLL‐IPI factors ß_2_‐microglobulin, immunoglobulin heavy variable (IGHV) status, and *TP53* status each retained prognostic value for PFS. The 3‐year overall survival (OS) rates by CLL‐IPI risk groups were 100%, 96%, 93.9%, and 89.4%, respectively, with no differences between consecutive risk groups. Age, Binet stage, ß_2_‐microglobulin, and *TP53* status each retained prognostic value for OS. In chemoimmunotherapy patients (median observation time, 66.9 months), 3‐year PFS rates for CLL‐IPI risk groups were 78.1%, 51.4%, 40.1%, and 16.5%, respectively; corresponding 3‐year OS rates were 97.4%, 93.1%, 81.8%, and 57.3% [58].

**TABLE 1:** Outcome of CLL patients of different CLL‐IPI categories in the era of targeted agents.

	Targeted therapies (median observation time 40.5 months)		Chemoimmunotherapy (median observation time 66.9 months)	
CLL‐IPI category	PFS (% at 3 years)	OS (% at 3 years)	PFS (% at 3 years)	OS (% at 3 years)
Low‐risk	96.5	100	78.1	97.4
Intermediate‐risk	87.6	96	51.4	93.1
High‐risk	82.4	93.9	40.1	81.8
Very high‐risk	78.7	89.4	16.5	57.3

*Note*: Estimates for progression‐free survival (PFS) and overall survival (OS) as reported by Langerbeins et al. [58]. Please note that the indication to treat does *not* depend on the CLL‐IPI category; most patients with a low‐risk CLL‐IPI are not treated at diagnosis, as they do not have symptomatic disease.

A system for predicting the time to first treatment in patients with CLL with early, asymptomatic disease, the International Prognostic Score for Early‐stage CLL [IPS‐E] [59] uses three covariates, unmutated IGHV gene, absolute lymphocyte count higher than 15 × 10^9^/L, and presence of palpable lymph nodes to separate low‐risk, intermediate‐risk, and high‐risk patients with a 5‐year cumulative risk for treatment start of 8.4%, 28.4%, and 61.2%, respectively. The IPS‐E will be helpful to counsel patients with early‐stage CLL.

**Criteria for the initiation of therapy** as proposed by the iwCLL guidelines remain unchanged [5]. The decision to initiate treatment depends on the presence of active/symptomatic disease. Asymptomatic patients with early‐stage disease (Rai 0, Binet A) should be monitored without therapy unless they have evidence of rapid disease progression or until the disease becomes symptomatic. This conservative approach of a watch‐and‐wait strategy is strongly supported by evidence, as multiple controlled, prospective studies on treating patients with early‐stage disease have *not* shown a survival benefit so far, regardless of the type of therapy, that is, chemotherapy alone, chemoimmunotherapy, or BTK inhibitors such as ibrutinib [60, 61, 62, 63, 64, 65, 66]. Therefore, an early therapeutic intervention in asymptomatic CLL is currently not recommended.

In an attempt to generate a prognostic tool for patients with CLL treated with ibrutinib, Ahn et al. identified four relevant factors [67]: *TP53* aberration, prior treatment, beta_2_ microglobulin ≥ 5 mg/L, and lactate dehydrogenase > 250 U/L. These factors were used to create three prognostic subgroups with 3‐year survival rates of 63%, 83%, and 93%. The model remained significant when applied to treatment‐naive and relapsed/refractory cohorts individually. Richter's transformation occurred in 17% of the high‐risk group, and in no patient in the low‐risk group. Overall, these factors may identify patients at increased risk of ibrutinib failure.

When patients progress or present with **progressive or symptomatic/active disease**, treatment should be initiated. The iwCLL guidelines [5] define symptomatic or active disease by defined criteria listed in Table 2.

**TABLE 2:** Criteria to define symptomatic or active disease according to iwCLL guidelines [5].

(1)	Evidence of progressive marrow failure as manifested by the development of, or worsening of, anemia and/or thrombocytopenia. Cut‐off levels of Hb < 10 g/dL or platelet counts of < 100,000/μL are generally regarded as indications for treatment. However, it should be pointed out that in some patients platelet counts of < 100,000/μL may remain stable over a long period of time; this situation does not automatically require therapeutic intervention.
(2)	Massive (i.e., ≥ 6 cm below the left costal margin) or progressive or symptomatic splenomegaly.
(3)	Massive nodes (i.e., ≥ 10 cm in longest diameter) or progressive or symptomatic lymphadenopathy.
(4)	Progressive lymphocytosis with an increase of ≥ 50% over a 2‐month period, or lymphocyte doubling time (LDT) of less than 6 months. LDT can be obtained by linear regression extrapolation of absolute lymphocyte counts (ALC) obtained at intervals of 2 weeks over an observation period of 2–3 months; patients with initial blood lymphocyte counts of < 30.000/μL may require a longer observation period to determine the LDT. Factors contributing to lymphocytosis other than CLL (e.g., infections, steroid administration) should be excluded.
(5)	Autoimmune complications including anemia or thrombocytopenia poorly responsive to corticosteroids.
(6)	Symptomatic or functional extranodal involvement (e.g., skin, kidney, lung, spine).
(7)	Disease‐related symptoms as defined by any of the following:
	Unintentional weight loss ≥ 10% within the previous 6 months.
	Significant fatigue (i.e., ECOG PS 2 or worse; cannot work or unable to perform usual activities).
	Fevers ≥ 100.5°F or 38.0°C for 2 or more weeks without evidence of infection.
	Night sweats for ≥ 1 month without evidence of infection.

Hypogammaglobinemia, or monoclonal or oligoclonal paraproteinemia does not by itself constitute a basis for initiating therapy. However, it is recommended to assess the change in these protein abnormalities if patients are treated. Also, patients with CLL may present with a markedly elevated leukocyte count; however, leukostasis rarely occurs in patients with CLL. Therefore, the absolute lymphocyte count should not be used as the sole indicator for treatment.

## 4 Response Assessment

**FIGURE 1:**
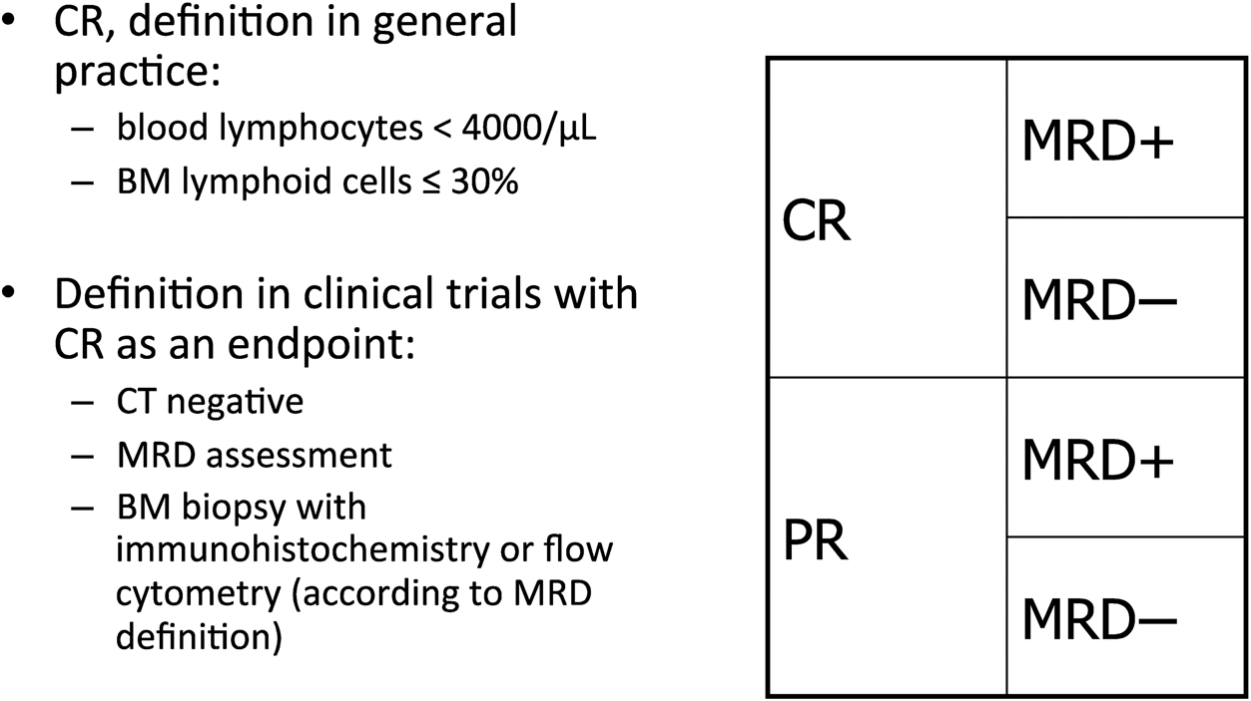
Definition of response in clinical trials, as proposed by the iwCLL [5]. Please note that the assessment of MRD is not always part of routine practice, but it may be used to determine the duration of therapy with targeted agents. MRD, minimal residual disease; CR, complete remission; PR, partial remission; MRD−, undetectable MRD; MRD+, detectable MRD.

The iwCLL guidelines give a detailed description of the assessment of the treatment response. A detailed overview of these response criteria is beyond the scope of this manuscript. In essence, the following response categories can be separated [5]: complete remission, partial remission, stable disease, and progression, as well as refractory disease. In addition, the assessment of minimal residual disease (MRD) is an additional and increasingly important category of response assessment, resulting in four different response categories (Figure 1).

### 4.1 Eradicating MRD

The use of sensitive multicolor flow cytometry, PCR, or next‐generation sequencing can detect minimal residual disease (MRD) in many patients who achieve a complete clinical response. Prospective clinical trials have provided evidence that therapies that lead to **undetectable MRD** (**uMRD**) usually improve long‐term clinical outcomes [68, 69, 70, 71, 72, 73, 74, 75, 76]. The value of MRD assessments has been compared to the evaluation of clinical response in CLL in 554 patients treated in two randomized trials of the German CLL Study Group (CLL8 and CLL10) [68]. Patients with uMRD in complete remission (CR), uMRD in PR, detectable MRD (dMRD) in CR, and dMRD in PR experienced a median PFS from a landmark at the end of treatment of 61, 54, 35, and 21 months, respectively. Interestingly, PFS did not differ significantly between uMRD CR and uMRD PR. In contrast to residual lymphadenopathy, persisting splenomegaly did not impact outcomes in patients with uMRD PR. In a retrospective, monocentric study, 536 patients with at least a partial response (PR) to various therapies between 1996 and 2007 received a bone marrow MRD assessment at the end of treatment [77]. MRD negativity correlated with both PFS and OS independent of the type and line of treatment, as well as known prognostic factors, including adverse cytogenetics. The greatest impact of achieving MRD negativity was seen in patients receiving frontline treatment, with 10‐year PFS of 65% versus 10% and 10‐year OS of 70% versus 30% for uMRD versus dMRD patients, respectively.

Techniques for assessing MRD have become well‐standardized, including six‐color flow cytometry (MRD flow), allele‐specific oligonucleotide PCR, and high‐throughput immune sequencing like ClonoSEQ, which can detect down to a level of less than one CLL cell in 10 000 leukocytes [78, 79]. A typical flow cytometry assay uses a core panel of six markers (CD19, CD20, CD5, CD43, CD79b, and CD81) [79]. Patients are considered to have uMRD if there are fewer than one CLL cell per 10 000 leukocytes in blood or marrow. While peripheral blood is typically assessed, some therapies may clear blood but leave marrow with detectable CLL, making marrow confirmation potentially relevant. Clinical trials should assess MRD as its absence has strong prognostic significance, and reports should clarify whether blood and/or marrow were analyzed, using the total number of patients in the treatment arm for reporting MRD‐neg proportions.

One approach to utilize MRD data for outcome predictions has been recently proposed with the Continuous Individualized Risk Index (CIRI) [80]. The CIRI can predict PFS and OS based on baseline CLL‐IPI and choice of therapy, but also on longitudinal knowledge like interim MRD or final MRD status, which allows for a refined prediction of outcomes. The algorithm was recently validated for a fixed‐duration therapy with venetoclax and obinutuzumab [81].

Collectively, there is overwhelming evidence to suggest that MRD quantification allows for improved PFS prediction in both patients who achieve a PR and CR, supporting its application in all responders. Although evaluation of MRD is still not generally recommended for routine clinical practice [5], I anticipate that MRD assessment will be highly relevant to guide the duration of therapies with novel inhibitors [82]. In my practice, I use MRD levels at increased frequency for the following treatment decisions: (A) Should I continue therapy in a high‐risk patient? (B) Should I stop therapy with targeted inhibitors? These questions are also being addressed in trials such as the FLAIR and the CLL18 protocols. These studies use uMRD as guidance to determine treatment duration [76].

## 5 Treatment of CLL

### 5.1 Description of Active Agents in CLL and Their Use as Monotherapy

#### 5.1.1 Cytostatic Agents

Monotherapy with **alkylating agents** has served as front‐line therapy for CLL, and chlorambucil was the therapeutic “gold standard” for several decades [63]. The advantages of chlorambucil are its low toxicity, low cost, and convenience as an oral drug; the major disadvantages are its low to non‐existent CR rate and some side effects that occur after extended use (prolonged cytopenia, myelodysplasia, and secondary acute leukemia). Today, the use of chlorambucil monotherapy is limited to countries without access to some of the newer agents and to achieve palliation in elderly or unfit patients after failure of targeted agents. It is reassuring that a recent analysis of more than 4135 patients from the Danish chronic lymphocytic leukemia registry diagnosed between 2008 and 2017 showed no major negative impact for first‐line therapy for any of the chemotherapies including chlorambucil monotherapy on OS, as second‐line therapies were able to rescue their lower efficacy [83].

Three **purine analogues** were investigated in CLL: fludarabine, pentostatin, and cladribine (2‐CdA). Fludarabine remains by far the best‐studied compound of the three in CLL. Fludarabine monotherapy produced more overall responses (OR) and complete remissions (CR) than other chemotherapies, like CHOP (cyclophosphamide, doxorubicin, vincristine, prednisone), CAP (cyclophosphamide, doxorubicin, prednisone), or chlorambucil, but did not improve OS [84, 85, 86, 87, 88, 89]. Similarly, cladribine monotherapy produces a higher CR rate than chlorambucil plus prednisone, without improving survival [90].

**Bendamustine** [91] was compared to chlorambucil and produced improved response rates, but showed greater toxicity and no survival benefit [92]. Bendamustine was also compared to fludarabine in 96 patients with relapsed CLL requiring treatment after one previous systemic regimen [93]. Overall and complete response rates were higher for bendamustine than for fludarabine, without improving OS. Collectively, these results established bendamustine as a potent single agent for the treatment of CLL.

#### 5.1.2 Monoclonal Antibodies

##### 5.1.2.1 Anti‐CD20 Antibodies

CD20 is an activated, glycosylated phosphoprotein expressed on the surface of mature B‐cells. The protein has no known natural ligand [94] and its function is unclear. It is suspected to act as a calcium channel in the cell membrane. As CD20 is expressed in most B‐cell malignancies, the introduction of the anti‐CD20 antibody rituximab in 1998 improved the treatment of most CD20‐positive non‐Hodgkin lymphomas including CLL [95]. Some newer CD20‐antibodies challenge rituximab [96, 97, 98].

**Rituximab**. In CLL, rituximab is less active as a single agent than in follicular lymphoma, unless very high doses are used [99, 100]. In contrast, combinations of rituximab with chemotherapy have proven to be very efficacious therapies for CLL.

**Ofatumumab** is a fully humanized antibody targeting a unique epitope on the CD20 molecule. It is no longer marketed for the treatment of B cell malignancies, despite interesting biological and clinical properties [101, 102].

**Obinutuzumab (GA101)**. The humanized and glycoengineered monoclonal antibody obinutuzumab is more active in vitro, inducing higher rates of apoptosis in B‐cells in comparison to rituximab [103]. The humanization of the parental B‐Ly1 mouse antibody and subsequent glycoengineering lead to higher affinity binding to a CD20 type II epitope, increased antibody‐dependent cellular cytotoxicity (ADCC), low complement‐dependent cytotoxicity (CDC) activity, and increased direct cell death induction [104]. The GAUGUIN trial, a Phase 1/2 trial, tested obinutuzumab monotherapy in patients with relapsed/refractory patients with CLL and confirmed that obinutuzumab was an active drug in CLL [105]: ORR was 62% (Phase 1) and 30% (Phase 2), respectively. Phase 2 median PFS was 10.7 months.

##### 5.1.2.2 Other Monoclonal Antibodies

**Alemtuzumab** is a recombinant, fully humanized, monoclonal antibody against the CD52 antigen. Monotherapy with alemtuzumab has produced response rates of 33%–53%, with a median duration of response ranging from 8.7 to 15.4 months, in patients with advanced CLL previously treated with alkylating agents who had failed or relapsed after second‐line fludarabine therapy [106, 107, 108]. Alemtuzumab has also proven effective in patients with high‐risk genetic markers [109, 110]. Therefore, alemtuzumab was a reasonable therapeutic option for relapsed patients with poor prognostic features. In a prospective randomized study, alemtuzumab was tested against chlorambucil and led to greater OR and CR rates, and longer PFS and OS [111]. In 2012, a strategic decision of Sanofi led to the withdrawal of the license of alemtuzumab for CLL, but the drug continues to be available as an approved agent to treat multiple sclerosis. However, upon the arrival of new oral agents, alemtuzumab lost its relevance in CLL therapy.

#### 5.1.3 Agents Targeting Signaling Pathways of CLL Cells and Their Microenvironment

Signaling through the B‐cell receptor (BCR) signaling plays an important role in the survival of CLL cells [112, 113]. Different aspects of the BCR have been recognized as prognostic marker in chronic lymphocytic leukemia, such as immunoglobulin heavy chain variable gene (IGHV) mutational status or stereotypy. Continuous or repetitive BCR signaling supports CLL cell survival (reviewed in [113]). This might explain why inhibition of BCR signaling is a potent strategy to treat CLL [114]. BCR signaling of CLL cells is transmitted by different tyrosine kinases, such as Bruton tyrosine kinase (BTK), Spleen tyrosine kinase (Syk), ZAP70, Src family kinases (in particular Lyn), and PI3K [114]. The advent of inhibitors of BTK or PI3K delta has revolutionized the therapy of B lymphoid malignancies. In addition, targeted deletion of BAKs such as Lyn and Btk in murine CLL models suggests that BAKs shape the dialogue between malignant B cells and the tumor microenvironment (TME) [33]. Since BAKs are expressed in multiple cell types, BAK inhibitors may disrupt the lymphoma‐supportive microenvironment [115]. This concept provides a mechanistic understanding of the typical clinical response to BAK inhibitor treatment, which is characterized by a transient increase of malignant B cells in the peripheral blood due to their mobilization from lymphoid homing compartments.

##### 5.1.3.1 PI3K Inhibitors

###### 5.1.3.1.1 Idelalisib

The PI3K pathway is constitutively activated in CLL and depends on the PI3K p110 δ isoform (PI3K‐ δ) isoform [116]. Idelalisib, an oral PI3Kδ‐isoform‐selective inhibitor, has shown good efficacy in patients with relapsed/refractory CLL, with nodal responses in 81% and an overall response rate in 72% of patients [117]. The most commonly observed grade ≥ 3 adverse events were pneumonia (20%), neutropenic fever (11%), and diarrhea (6%).

###### 5.1.3.1.2 Duvelisib

Duvelisib is an oral inhibitor of both the delta and gamma isoforms of PI3K. A Phase 1 trial included 55 relapsed/refractory patients with CLL, 56% showed an ORR [118]. In the Phase 3 DUO trial, patients with relapsed/refractory CLL were randomized to receive duvelisib 25 mg twice daily or ofatumumab. Median PFS was 13 months with duvelisib compared to 10 months with ofatumumab [119]. The most frequent toxicities of duvelisib include hematologic toxicities, elevated transaminases, and diarrhea, as well as PJP and CMV infections. This suggests that the toxicity profile is similar to idelalisib. Duvelisib was approved in the US for treating CLL after at least two prior lines of therapy.

###### 5.1.3.1.3 Umbralisib

Umbralisib is a dual inhibitor of PI3Kdelta and CK1ε. It has shown good efficacy in relapsed/refractory CLL with an ORR of 62% in combination with a CD20 antibody, ublituximab [120]. While the rates of transaminitis (2%–3%) or diarrhea (3%–10%) seem lower, the other side effects are similar to idelalisib or duvelisib [121]. The Phase 3 UNITY study investigated umbralisib in combination with ublituximab (U2 regimen) in treatment‐naïve and relapsed/refractory CLL. It reported a median PFS of 32 months in treatment‐naïve CLL [122]. However, transaminitis, diarrhea, and pneumonitis occurred more frequently than in the control arm with chlorambucil. The license application for the ublituximab plus umbralisib combination was withdrawn when findings from the UNITY trial revealed a growing imbalance in OS [123].

The reports on the side effects of PI3K inhibitor treatment have strongly reduced the use and stopped the further development of this class of agents for the treatment of CLL/SLL [124].

##### 5.1.3.2 BTK Inhibitors

Bruton tyrosine kinase (BTK) leads to downstream activation of B‐cell survival pathways such as NF‐κB and MAP kinases via Src family kinases [125]. These pathways play a relevant role in the signal transduction of the BCR. Inhibitors of BTK have become a new class of very active therapeutic agents in B‐cell malignancies [126].

###### 5.1.3.2.1 Ibrutinib

Ibrutinib is an orally active, small‐molecule BTK inhibitor that induces apoptosis in B‐cell lymphomas and CLL cells [125]. In one of the first trials, 56 patients with relapsed or refractory B‐cell lymphoma and CLL received escalating oral doses of ibrutinib, at two schedules: one, 28 days on, 7 days off; and two, once‐daily continuous dosing. The ORR in 50 evaluable patients was 60%, including 16% CR. The median PFS in all patients was 13.6 months [127]. The most relevant treatment‐related side effects were viral infections.

In a study of 85 patients with relapsed or refractory CLL or SLL, ibrutinib showed promising results [128]. Patients experienced mostly Grade 1 or 2 side effects such as transient diarrhea, fatigue, and upper respiratory tract infection. The overall response rate was 71%, with additional patients showing partial responses with lymphocytosis. The estimated PFS rate at 26 months was 75%, and the OS rate was 83%. These results indicate that ibrutinib can provide durable remissions in CLL/SLL patients with relapsed, refractory, or high‐risk disease.

Ahn et al. reported that ibrutinib showed promise in treating CLL patients with *TP53* alterations, with 61% PFS and 79% OS at 6 years [129]. At 6 years of treatment, the estimated percentage of patients with PFS and OS was 61% and 79%, respectively. However, *TP53* aberrations remain an unfavorable prognostic factor with continuous BTK inhibitor monotherapy when compared to other factors [67, 130].

Ibrutinib was compared to ofatumumab in a Phase 3 study with 391 patients with relapsed or refractory CLL or SLL [131]. Ibrutinib significantly improved PFS compared to ofatumumab, with a PFS rate of 88% at 6 months. Ibrutinib also significantly improved OS, with a 12‐month OS rate of 90% compared to 81% for ofatumumab.

The RESONATE‐2 trial established ibrutinib monotherapy as a first‐line option in patients with CLL by demonstrating a significant improvement in survival [132]. The results were impressive, especially for patients with CLL with high‐risk genetics. However, as chlorambucil monotherapy was no longer considered an appropriate standard, additional trials compared ibrutinib to more potent therapies. Woyach et al. compared ibrutinib alone or in combination with rituximab to a first‐line therapy with bendamustine and rituximab (BR) for patients with CLL ≥ 65 years of age [130]. The study showed a superior PFS for ibrutinib and IR compared to BR. The addition of rituximab to ibrutinib did not result in prolonged PFS. There was no significant PFS advantage observed in patients with mutated IGHV. No OS benefit was seen for any of the arms.

An Italian CLL network conducted a study on CLL patients without *TP53* disruption treated with ibrutinib or obinutuzumab‐chlorambucil as first‐line therapy MRD [133]. The study found that while the overall response rates between the two treatments were similar, more complete remissions were achieved with obinutuzumab‐chlorambucil. After a 30‐month follow‐up, it was observed that ibrutinib provided better PFS and time to next treatment (TTNT), especially in patients with unmutated IGHV. The authors concluded that continuous ibrutinib treatment provided better disease control, while a fixed‐duration obinutuzumab‐based treatment showed significant clinical and economic benefits in M‐CLL patients and those achieving an uMRD [133].

As **acquired treatment resistance to ibrutinib** therapy was observed in an increasing number of patients, whole‐exome sequencing studies were performed and identified a cysteine‐to‐serine mutation in BTK at the binding site of ibrutinib and three distinct mutations in PLCgamma2 [134]. The C481S mutation of BTK results in a protein that is only reversibly inhibited by ibrutinib. The R665W and L845F mutations in PLCgamma2 are gain‐of‐function mutations leading to the autonomous activity of B cell receptor‐stimulated pathways. A study on 308 ibrutinib‐treated patients found that patients who discontinued therapy due to disease progression had poor outcomes [135]. Richter's transformation occurred early, with a median survival of only 3.5 months, while CLL progressions occurred later, with a median survival of 17.6 months. Mutations in BTK or PLCgamma2 were found in patients with CLL progression, which were absent before treatment. A later analysis of the same institution with a median follow‐up time of 3.4 years showed a cumulative incidence of progression at 4 years of 19% [136]. Baseline karyotypic complexity, presence of del(17)(p13.1), and age less than 65 years were risk factors for progression. Among patients who experienced relapse, acquired mutations of BTK or PLCG2 were found in 85%. These mutations were detected at an estimated median of 9.3 months before relapse. A prospective examination of a group of 112 patients found that eight patients experienced relapse, with acquired resistance mutations occurring before relapse. Resistance mutations were detected in an additional eight patients who did not meet the criteria for clinical relapse.

Patients treated with ibrutinib show a distinct toxicity pattern. This particularly relates to off‐target effects that lead to an increased risk of cardiac arrhythmia, in particular atrial fibrillation (AF), cardiac failure, bleeding, and hypertension [137, 138, 139]. The occurrence of AF, which occurs typically in elderly patients with CLL regularly necessitates therapeutic anticoagulation, which potentially increases the risk of bleeding events. This cardiac toxicity is not associated with AF‐associated thromboembolism or acute myocardial infarction [140].

###### 5.1.3.2.2 Acalabrutinib

Acalabrutinib is a highly selective, irreversible BTK inhibitor with improved safety and efficacy compared to ibrutinib. In a Phase 1–2 study, 61 patients with relapsed CLL were treated with acalabrutinib at various doses with promising results portion [141]. A follow‐up analysis confirmed the effectiveness and safety profile in 134 patients with relapsed/refractory CLL or SLL receiving acalabrutinib 100 mg twice daily, with most adverse events being mild or moderate [142], mostly commonly diarrhea (52%) and headache (51%). Grade ≥ 3 AEs (observed in ≥ 5% of patients) were neutropenia (14%), pneumonia (11%), hypertension (7%), anemia (7%), and diarrhea (5%). Atrial fibrillation and major bleeding AEs (all grades) occurred in 7% and 5% of patients, respectively. The overall response rate was 94%, and the estimated 45‐month PFS was 62%. BTK mutations were detected in six of nine patients at relapse.

Another study with 99 treatment‐naive CLL patients showed an overall response rate of 97% after a median follow‐up of 53 months [143]. The overall response rate was 97% (7% complete responses), with similar outcomes among all prognostic subgroups. Because of improved trough BTK occupancy with twice‐daily dosing, all patients were transitioned to 100 mg twice daily. Serious adverse events were reported in 38% of patients, with 6% discontinuing treatment due to adverse events. Grade ≥ 3 events of special interest included infection (15%), hypertension (11%), bleeding events (3%), and atrial fibrillation (2%).

Phase 2 studies of acalabrutinib were also conducted in patients with relapsed/refractory CLL intolerant to ibrutinib [144, 145]. Sixty patients were treated with acalabrutinib, with an overall response rate of 73% and three patients achieved a complete remission [144]. The most common adverse events with acalabrutinib were diarrhea, headache, contusion, dizziness, upper respiratory tract infection, and cough. The most common reasons for acalabrutinib discontinuation were progressive disease and adverse events. Similar results were obtained in another Phase 2 study, which confirmed good tolerance and high response to acalabrutinib after ibrutinib intolerance [145] Together, these studies indicate that acalabrutinib is beneficial for some patients who are ibrutinib intolerant.

Thereafter, acalabrutinib was studied in a Phase 3 trial involving 310 patients with relapsed/refractory CLL [146]. The study compared acalabrutinib monotherapy to idelalisib plus rituximab [I‐R] or bendamustine plus rituximab (BR), depending on the investigator's choice. Of the 310 patients, 155 received acalabrutinib monotherapy, while the other 155 received the investigator's choice (I‐R for 119 patients and BR for 36 patients). After a median follow‐up of 16.1 months, it was found that the median PFS was significantly longer with acalabrutinib monotherapy (PFS not reached) compared to the investigator's choice (16.5 months). Serious adverse events were reported in 29% of patients treated with acalabrutinib monotherapy, 56% with I‐R, and 26% with BR. The study also noted that deaths occurred in 10% (15 out of 154), 11% (13 out of 118), and 14% (5 out of 35) of patients receiving acalabrutinib monotherapy, I‐R, and BR, respectively. Based on these results, acalabrutinib monotherapy was approved for treating relapsed/refractory CLL.

To test whether the higher specificity of acalabrutinib leads to clinically meaningful reductions of toxicity, the ELEVATE‐RR study was performed [147]. In this Phase 3 trial, patients with high‐risk CLL were given either ibrutinib or acalabrutinib. Acalabrutinib showed a favorable toxicity profile compared with ibrutinib, with lower rates of atrial fibrillation/flutter, bleeding, and hypertension.

###### 5.1.3.2.3 Zanubrutinib

Like acalabrutinib, zanubrutinib is a second‐generation, covalent BTK inhibitor with higher specificity and less off‐target inhibition than ibrutinib. It was initially tested in a Phase 1 study of various B cell malignancies [148]. Additional data were gained from a Phase 2 trial using zanubrutinib 160 mg twice daily in 91 Chinese patients with relapsed CLL [149]. The study reported an ORR of 82%–86% in patients with low‐ and high‐risk CLL. While bleeding‐associated AEs, including petechiae or contusions, were quite common (35%), atrial fibrillation was not observed. To perform a head‐to‐head comparison between ibrutinib and zanubrutinib, the ALPINE Phase 3 study included patients with relapsed/refractory CLL, who were treated with zanubrutinib or ibrutinib until non‐tolerance or disease progression [150]. After a median follow‐up of 29.6 months, the results indicated that zanubrutinib was superior to ibrutinib concerning PFS. At the 24‐month mark, the investigator‐assessed PFS rates were 78.4% for the zanubrutinib group and 65.9% for the ibrutinib group. Among patients with a del(17p), a *TP53* mutation, or both, those treated with zanubrutinib demonstrated longer PFS compared to those receiving ibrutinib. Additionally, PFS rates consistently favored zanubrutinib across other major subgroups. The safety profile of zanubrutinib was also better than that of ibrutinib, with fewer adverse events leading to treatment discontinuation and a lower incidence of cardiac events, including those that resulted in treatment discontinuation or death.

###### 5.1.3.2.4 Pirtobrutinib

Pirtobrutinib is a highly selective, but reversible (non‐covalent) BTK inhibitor, which also has activity in patients with C481S mutation of BTK. In a recent Phase 1/2 trial, 323 patients with previously treated B‐cell malignancies were treated with pirtobrutinib across seven dose levels (25, 50, 100, 150, 200, 250, and 300 mg once per day) [151]. No dose‐limiting toxicities were reported, and the maximum tolerated dose was not reached. The study continued with a recommended Phase 2 dose of 200 mg/d. Adverse events occurring in at least 10% of 323 patients were fatigue (65 [20%]), diarrhea (55 [17%]), and contusion (42 [13%]). The most common adverse event of Grade 3 or higher was neutropenia (32 [10%]). Of particular importance, the study did not report any Grade 3 atrial fibrillation or flutter. A Grade 3 hemorrhage was observed in one patient in the setting of mechanical trauma. Only five (1%) patients discontinued treatment due to a treatment‐related adverse event. In 121 CLL or SLL patients who were evaluable for efficacy and had received a covalent BTK inhibitor before the study, the ORR with pirtobrutinib was 62%. The overall response rate was similar in patients with CLL with previous covalent BTK inhibitor resistance (53 [67%] of 79), covalent BTK inhibitor intolerance (22 [52%] of 42), BTK C481‐mutant (17 [71%] of 24) and BTK wild‐type (43 [66%] of 65) disease. The results indicate that non‐covalent BTK inhibitors like pirtobrutinib suitable agents for patients with intolerance of or resistance to conventional BTK inhibitors.

###### 5.1.3.2.5 BTK Degraders

BTK degraders, including proteolysis‐targeting chimeras (PROTACs), work by harnessing the cell's natural protein degradation machinery to selectively target and degrade proteins and have gained relevance in the field of hematological malignancies [152]. Unlike traditional inhibitors that merely block the active site of the enzyme, BTK degraders may bind to BTK and recruit E3 ubiquitin ligases, which tag BTK for destruction by the proteasome (reviewed in [152, 153]). This results in a more complete and sustained reduction of BTK levels. BTK degraders have advantages over traditional BTK inhibitors. They can target both wild‐type and mutant forms of BTK, including those with the C481S mutation, a common resistance mechanism to covalent BTK inhibitors like ibrutinib [152]. Degrading the entire BTK protein can potentially reduce off‐target effects and improve safety. However, BTK degraders are still in early clinical development, and challenges remain. Managing treatment‐emergent adverse events is a primary concern.

There are several ongoing clinical trials for BTK degraders for patients with CLL and other B‐cell malignancies. NX‐5948 is an oral BTK degrader currently investigated in patients with relapsed/refractory CLL and non‐Hodgkin's lymphoma [154]. Early findings in a heavily pre‐treated population of patients with CLL and NHL indicated that NX‐5948 was safe and well tolerated and has clinical activity, supporting the continuation of its development in CLL and NHL. NX‐5948 also exhibited dose‐proportional pharmacokinetics, resulting in rapid, robust, and sustained BTK degradation [154]. BGB‐16673 is another potent BTK degrader being tested in a Phase 1 trial [155]. In a phase I trial with 26 patients with various B cell malignancies including 10 CLL encouraging results were reported regarding the efficacy. Treatment‐emergent AEs were reported by 88.5% of pts., the most common being contusion, pyrexia, neutropenia, and lipase increases. No hypertension or atrial fibrillation was observed. Of 18 response‐evaluable pts., 12 (67%) responded, with responses starting at the lowest dose level [155].

A BTK and IKZF1/3 degrader, NX‐2127, has been shown to bind and degrade mutant BTK proteoforms, effectively blocking BCR signaling [156]. In a Phase 1 trial for patients with relapsed/refractory B cell malignancies, including CLL, 47 patients received daily doses of 100, 200, or 300 mg [157]. Among those, 29 had CLL/small lymphocytic lymphoma, most of whom had previously been treated with BTK (100%) and BCL2 inhibitors (76%). Common grade ≥ 3 treatment‐emergent adverse events included neutropenia (38.3%), hypertension (14.9%), and anemia (12.8%). Treatment discontinuation was primarily due to progressive disease (25.5%) and adverse events (21.3%). NX‐2127 exhibited dose‐dependent pharmacokinetics with a half‐life of 2–4 days and consistent BTK degradation. Among evaluable CLL patients, results included 9 partial responses, 11 stable diseases, and 4 progressive disease cases at the data cut‐off.

#### 5.1.4 Lenalidomide

Lenalidomide is a thalidomide analog used in the treatment of myelodysplastic syndrome and multiple myeloma. It showed encouraging results in the treatment of high‐risk patients with CLL including carriers of a del(17p) [158]. In 58% of patients, it causes tumor flare reactions, a sensation of heat, and burning in the lymph nodes [159, 160]. The overall response rate of lenalidomide monotherapy in CLL varied between 32% and 54% [160, 161]. Long‐term outcomes were reported in a study reported at a median follow‐up of 4 years [162]; long‐term responders to lenalidomide had a median OS of 82% and showed improvements in immunoglobulin levels and T‐cell numbers.

Lenalidomide was also investigated as maintenance therapy in high‐risk CLL. In one trial, patients with CLL with at least a partial response after chemoimmunotherapy were eligible, if they had the detectable MRD combined with unmutated IGHV or *TP53* gene alterations [163]. While lenalidomide maintenance prolonged the PFS, it carried the risk of transformation to acute lymphoblastic leukemia [164]. Similar observations were made in a Phase 3 study of lenalidomide versus placebo maintenance following second‐line therapy, and no OS benefit was observed [165]. Therefore, lenalidomide is *not* recommended as a maintenance therapy for CLL.

#### 5.1.5 BCL‐2 Inhibitors

Proteins in the B cell lymphoma 2 (Bcl‐2) family are key regulators of the apoptotic process [166]. The Bcl‐2 family comprises proapoptotic and prosurvival proteins. Shifting the balance toward the latter is an established mechanism whereby cancer cells evade apoptosis. Bcl‐2, the founding member of this protein family, is encoded by the BCL2 gene initially described in follicular lymphoma as a protein in translocations involving chromosomes 14 and 18 [167].

##### 5.1.5.1 Venetoclax

Venetoclax is a BH3‐mimetic drug designed to block the function of the Bcl‐2 protein [168]. Venetoclax inhibits the growth of BCL‐2‐dependent tumors. A single oral dose of venetoclax in three patients with refractory chronic lymphocytic leukemia resulted in tumor lysis within 24 h [168]. Therefore, a dose escalation scheme was installed to prevent these incidents [169], with a weekly dose ramp‐up schedule (20, 50, 100, 200, 400 mg) over 4–5 weeks. Thereafter, patients should take daily 400 mg continuously dosing until disease progression or side effects occur [170]. In a pivotal Phase 1/2 trial, 56 patients received venetoclax in one of eight dose groups that ranged from 150 to 1200 mg per day [171]. In an expansion cohort, 60 additional patients were treated with venetoclax using a weekly stepwise ramp‐up in doses up to 400 mg per day. The majority of the patients had received multiple previous treatments, and 89% had poor prognostic clinical or genetic features. Venetoclax was active at all dose levels. Clinical tumor lysis syndrome occurred in 3 of 56 patients in the dose‐escalation cohort. After adjustments to the dose‐escalation schedule, no clinical tumor lysis syndrome occurred. Other side effects included mild diarrhea, upper respiratory tract infections, nausea, and Grade 3 or 4 neutropenias. Among the 116 patients who received venetoclax, 92 (79%) had a response. Response rates ranged from 71% to 79% among patients in subgroups with an adverse prognosis. Complete remissions occurred in 20%, including 5% uMRD remissions. The 15‐month PFS estimate for the 400‐mg dose groups was 69%.

In a trial with 107 patients with relapsed or refractory del(17p) CLL, venetoclax monotherapy showed an overall response rate of 79.4% at a median follow‐up of 12.1 months [170]. The most common Grade 3–4 adverse events were neutropenia (40%), infection (20%), anemia (18%), and thrombocytopenia (15%). Serious adverse events occurred in 55% of patients, with the most common being pyrexia, autoimmune hemolytic anemia, pneumonia, and febrile neutropenia. Eleven patients died within 30 days of the last dose of venetoclax; seven due to disease progression and four from an adverse event (none assessed as treatment‐related). Overall, venetoclax monotherapy is considered active and well‐tolerated in patients with relapsed or refractory high‐risk CLL.

#### 5.1.6 Checkpoint Inhibitors

Preclinical evidence suggests that the **programmed death 1 (PD‐1) pathway** is critical for inhibiting the immune surveillance of CLL. Therefore, a Phase 2 trial was performed with **pembrolizumab**, a humanized PD‐1‐blocking antibody in relapsed and transformed CLL [172]. Twenty‐five patients (16 relapsed CLL and 9 Richter transformations) were enrolled, and 60% received prior ibrutinib. Objective responses were observed in 4 out of 9 RT patients (44%) and 0 out of 16 patients with CLL (0%). Treatment‐related Grade 3 or above adverse events were reported in 15 (60%) patients and manageable. Analyses of pretreatment tumor specimens revealed increased expression of PD‐L1 and a trend of increased expression of PD‐1 in the microenvironment in patients who had confirmed responses. The results of this study suggest a benefit of PD‐1 blockade in patients with RT. As the efficacy of checkpoint inhibitor monotherapy does not appear sufficiently durable [173], several studies have tested combinations of checkpoint inhibitors with kinase inhibitors for RT therapy (see below).

#### 5.1.7 Cart Cells

An initial report using a lentiviral vector expressing a **chimeric antigen receptor** (**CAR**) with specificity for the B‐cell antigen CD19, coupled with CD137 (a costimulatory receptor in T cells [4‐1BB]) and CD3‐zeta (a signal‐transduction component of the T‐cell antigen receptor) signaling domains showed a very impressive efficacy [174]. A low dose (approximately 1.5 × 10^5^ cells per kilogram of body weight) of autologous CAR‐modified T cells reinfused into a patient with refractory CLL expanded to a level that was more than 1000 times as high as the initial engraftment level in vivo, with delayed development of a tumor lysis syndrome and subsequent CR.

An anti‐CD19 CAR‐T cell therapy was applied to 24 patients with CLL who had previously received ibrutinib [175]. Patients received lymphodepleting chemotherapy and anti‐CD19 CAR‐T cells at one of three dose levels (2 × 10^5^, 2 × 10^6^, or 2 × 10^7^ CAR‐T cells/kg). Four weeks after CAR‐T cell infusion, the overall response rate was 71% (17 of 24). In 19 of these patients who were restaged, the overall response rate 4 weeks after infusion was 74% (CR, 4/19, 21%; PR, 10/19, 53%), and 15/17 patients (88%) with marrow disease before CAR‐T cells had no disease by flow cytometry after CAR‐T cells, and seven (58%) had no malignant IGH sequences detected in the bone marrow. The absence of the malignant IGH clone in the marrow of patients with CLL who responded by IWCLL criteria was associated with 100% PFS and OS (median 6.6 months follow‐up).

More recently, a longer follow‐up of anti‐CD19 CART cell therapy was reported in patients with relapsed or refractory CLL [176]. Between 2013 and 2016, 42 patients with relapsed or refractory CLL were enrolled in this study and 38 were infused with anti‐CD19 CART cells (CART‐19). Of these, 28 patients were initially randomly assigned to receive a low (5 × 10^7^) or high (5 × 10^8^) dose of CART‐19. Twenty‐four patients were evaluable for response assessment. After an interim analysis, 10 additional patients received the selected, high dose, and of these, eight were evaluable for response. Patients were followed for a median of 31.5 months. At 4 weeks, the complete and overall responses for the 32 evaluable patients were 28% and 44%, respectively. The median OS for all patients was 64 months; there was no statistically significant difference between low‐ and high‐dose groups (*p* = 0.84). Regardless of dose, prolonged survival was observed in patients who achieved a CR versus those who did not (*p* = 0.035), with median OS not reached in patients with CR versus 64 months in those without CR. The median PFS was 40.2 months in patients with CR and 1 month in those without a CR. Toxicity was comparable in both dose groups. The results illustrate that attainment of a CR after CART‐19 infusion is associated with longer OS and PFS in patients with relapsed CLL.

The TRANSCEND‐CLL 004 study tested lisocabtagene maraleucel (liso‐cel) in patients with relapsed or refractory chronic lymphocytic leukemia or small lymphocytic lymphoma [177]. One hundred and seventeen patients received liso‐cel at one of two dose levels. The study showed a complete response or remission rate of 18% at the higher dose level. However, a relevant number of Grade 3 cytokine release syndrome and neurological events were reported. Among 51 deaths in the study, 43 occurred after liso‐cel infusion, of which five were due to treatment‐emergent adverse events (within 90 days of liso‐cel infusion). One death was related to liso‐cel (caused by a macrophage activation syndrome).

Overall, these observations highlight the potential of CD19 CAR‐T cells in CLL, but additional clinical studies need to be performed before recommending this modality on a broader basis or outside of clinical trials for relapsed or refractory patients with CLL.

### 5.2 Combination Therapies

One of the key principles of designing more efficient treatments of CLL has been the use of drug combinations with synergistic or at least additive efficacy but non‐overlapping toxicity. This principle has recently been expanded to the use of targeted agents that usually do not have identical toxicity profiles and hold the promise of long‐term control of CLL following a short, fixed‐duration treatment with the most potent inhibitors [82, 178]. The subsequent sections will summarize the most relevant results obtained with different drug combinations in CLL.

#### 5.2.1 Chemotherapy Combinations

Since purine analogs and alkylating agents have different mechanisms of action and partially non‐overlapping toxicity profiles, it seemed logical to combine the two modalities for achieving synergistic effects. Preclinical studies demonstrated that exposure of CLL cells to fludarabine and cyclophosphamide resulted in synergistic cytotoxicity [179]. Fludarabine has been evaluated in a variety of combination regimens. The combination of fludarabine with another purine analog, cytarabine, appeared to be less effective than fludarabine alone, while the combination of fludarabine with chlorambucil or prednisone increased the hematological toxicity but not the response rate [86, 180]. The most thoroughly studied combination chemotherapy for CLL is **fludarabine plus cyclophosphamide** (**FC**) which generated promising results in Phase 2 trials [180, 181]. A Phase 2 study of cladribine in combination with cyclophosphamide also demonstrated activity in advanced CLL, but the results seemed inferior to FC [182].

Later, three randomized trials showed that FC combination chemotherapy improved the CR and OR rate and PFS as compared to fludarabine monotherapy [183, 184, 185]. The rate of severe infections was not significantly increased by the FC combination despite a higher frequency of neutropenias. A re‐analysis of the CLL4 trial of the GCLLSG suggested that the first‐line treatment of patients with CLL with FC combination may improve the OS of low to intermediate‐risk patients with CLL (i.e., patients *not* exhibiting a del(17p) or *TP53* mutation). A Polish study group compared 2‐CdA alone to 2‐CdA combined with cyclophosphamide (CC) or to cyclophosphamide and mitoxantrone (CMC) in 479 cases with untreated progressive CLL [186]. Surprisingly, the CC or CMC combination therapies did not produce any benefit in terms of PFS or response rates when compared to 2‐CdA alone.

#### 5.2.2 Chemoimmunotherapy Using Monoclonal Antibodies Binding to CD20

In preclinical studies, rituximab and fludarabine showed synergy [187], leading to Phase 2 trials. Results of these trials showed high response rates and improved progression‐free and OS when rituximab was combined with fludarabine [188, 189, 190]. The MD Anderson Cancer Center conducted a phase II trial on 300 patients with previously untreated CLL, using **rituximab combined with fludarabine and cyclophosphamide** (**FCR**). The trial achieved an overall response rate of 95%, with CR in 72%, nodular PR in 10%, PR due to cytopenia in 7%, and PR due to residual disease in 6% [191]. Six‐year overall and failure‐free survival was 77% and 51%, respectively. The median time to progression was 80 months.

The GCLLSG conducted the randomized CLL8 trial with 817 patients [11]. The trial compared the effectiveness of FCR versus FC. It was the first prospective, randomized trial demonstrating a survival benefit for a first‐line therapy in CLL. FCR induced a higher OR rate than FC (92.8 vs. 85.4%) and more CR (44.5 vs. 22.9). PFS at 2 years was 76.6% in the FCR arm and 62.3% in the FC arm. Three years after randomization, 65% of patients in the FCR group were free of progression compared with 45% in the chemotherapy group; 87% were alive versus 83%, respectively. FCR was more frequently associated with Grade 3 and 4 neutropenia, while other side effects, including severe infections, were not increased. However, FCR did not improve the survival of patients with a del(17p). This trial established FCR as the standard first‐line therapy for fit CLL patients.

Updates of the CLL8 trial and the MD Anderson patient cohort demonstrated a very good outcome upon FCR therapy for specific subgroups, in particular in patients with a mutated IGVH, del(13q), trisomy 12 or del(11q), or patients achieving an uMRD remission [192, 193]. These patients seemed to achieve very durable remissions and a very good OS rate following FCR treatment. An extended follow‐up of the MD Anderson trial was published recently, with a median observation time of 19.0 years [194]. In this report, the median PFS for patients with IGHV‐M was 14.6 years versus 4.2 years for patients with unmutated IGHV. Disease progression beyond 10 years was uncommon. Only 16 of 94 (17%) patients in remission at 10 years subsequently showed a disease progression. The results show that FCR can achieve a functional cure of CLL in a significant fraction of patients.

Similarly good results were obtained in a trial comparing FCR to FC therapy in the second line [195], where FCR induced higher response rates and longer remissions than FC. A dose‐modified FCR‐Lite regimen aimed to decrease the toxicity of the FCR regimen [196]. This regimen reduced the dose of fludarabine and cyclophosphamide and increased the dose of rituximab. The CR rate was 77% for 50 previously untreated patients with CLL, with an OR rate of 100%. Grade 3/4 neutropenia was documented in only 13% of cycles, which was lower than observed with the standard FCR regimen.

As bendamustine became popular, Phase 2 studies investigated the combination of **bendamustine with rituximab**. In 81 patients with relapsed CLL, the overall response rate was 59.0%, with a median event‐free survival of 14.7 months [197]. Severe infections occurred in 12.8% of patients, and Grade 3 or 4 hematologic toxicities were documented. The BR regimen was also investigated as first‐line therapy in 117 patients with CLL [198]. The overall response rate was 88.0% with a complete response rate of 23.1% and a partial response rate of 64.9%. After a median observation time of 27.0 months, median event‐free survival was 33.9 months, and 90.5% of patients were alive. Grade 3 or 4 severe infections occurred in 7.7% of patients. Grade 3 or 4 adverse events for neutropenia, thrombocytopenia, and anemia were documented in 19.7%, 22.2%, and 19.7% of patients, respectively.

In the CLL10 study, FCR showed superior outcomes compared to BR, with longer PFS and more patients achieving minimal residual disease (MRD) negativity [199]. However, FCR was associated with higher rates of severe neutropenia and infections, especially in patients over 65 years. FCR remained the standard therapy for very fit patients with CLL, while BR was considered an alternative regimen for elderly fit patients.

Alemtuzumab or mitoxantrone was added to FCR to improve its efficacy, but both regimens resulted in limited improvements and increased toxicity [200, 201]. Similarly, replacing fludarabine with pentostatin in the FCR regimen did not show significant improvements [202]. Other combinations like cladribine with rituximab, methylprednisolone plus rituximab followed by alemtuzumab, or rituximab plus alemtuzumab also did not result in higher efficacy compared to FCR.

The CLL11 protocol from the GCLLSG studied chemoimmunotherapies with anti‐CD20 antibodies and chlorambucil (Clb) in untreated CLL patients with comorbidities [203]. The study was motivated by promising Phase 2 trial results using Clb‐R [204, 205] and the run‐in phase of CLL11 involving a **combination of chlorambucil with obinutuzumab** (Clb‐Obi) [206]. In the CLL11 trial, 781 untreated CLL patients with a CIRS score > 6 or a creatinine clearance of 30–69 mL/min were assigned to Clb, Clb‐Obi, or Clb‐rituximab (Clb‐R). The median patient age was 73 years, with a baseline creatinine clearance of 62 mL/min and a CIRS score of 8. Both Clb‐Obi and Clb‐R significantly raised response rates and extended PFS. Clb‐Obi also improved OS compared to Clb alone. Compared to Clb‐R, it yielded longer PFS and higher complete response rates. In a later follow‐up, the CLL11 study showed a significant OS advantage for obinutuzumab over rituximab [207].

Similarly, the anti‐CD20 antibody ofatumumab in combination with Clb improved outcomes when compared with Clb [208]. Overall, combining anti‐CD20 antibodies with chemotherapy enhanced outcomes in CLL patients with comorbidities, with obinutuzumab showing superiority over rituximab.

The anti‐CD52 antibody alemtuzumab was tested in combination with chemotherapy, in particular with fludarabine. While alemtuzumab in combination with FC (FCA) or fludarabine (FA) showed a higher efficacy, the treatment‐related toxicity or mortality was enhanced [209, 210]. In light of the recent advances in targeted agents used for CLL, these combinations are no longer used.

#### 5.2.3 Combinations Using Lenalidomide

The combination of **lenalidomide and rituximab** seems to increase the response rate without increasing the toxicity, even in patients with del(17p) and/or unmutated IGHV status. In a Phase 2 trial, 59 patients with relapsed or refractory CLL received a combination of lenalidomide and rituximab [211]. In this trial, oral daily therapy with 10 mg lenalidomide was started on day 9 of cycle one. Rituximab was administered at 28‐day intervals for up to 12 cycles; lenalidomide could continue indefinitely if patients benefitted clinically. The overall response rate was 66%. The most frequent Grade 3 or 4 toxicity was neutropenia (73% of patients), and 24% experienced Grade 3 to 4 infections or febrile episodes. In essence, this combination is a helpful alternative for patients with refractory CLL.

Combinations using drug triplets such as lenalidomide, rituximab, and fludarabine [212, 213, 214] or bendamustine, rituximab, and lenalidomide resulted in relatively high toxicity and disappointing response rates [215].

#### 5.2.4 Combinations Using Idelalisib

The PI3K delta inhibitor, idelalisib, was investigated in a Phase 3 study in combination with rituximab versus rituximab plus placebo [216]. The trial included 220 patients with decreased renal function, previous therapy‐induced myelosuppression, or major coexisting illnesses. Patients receiving idelalisib versus those receiving a placebo had improved rates of responses and survival at 12 months. These results led to the approval of idelalisib and rituximab for patients with relapsed CLL. The long‐term efficacy and safety of this treatment was reported in 110 patients [217]. The idelalisib/rituximab group had a median PFS of 20.3 months with an ORR of 85.5%. The median OS was 40.6 months. Prolonged exposure to idelalisib increased the incidence of diarrhea, colitis, and pneumonitis.

In a study with 261 patients, idelalisib in combination with ofatumumab showed a median PFS of 16.3 months compared to 8.0 months with ofatumumab alone [218]. Serious infections, including pneumonia and sepsis, were more common in the idelalisib‐ofatumumab group. Due to these findings, the FDA issued a warning regarding the toxicities associated with idelalisib therapy, for which patients should be monitored [219]. These toxicities include fatal and/or serious hepatotoxicity (in 16%–18% of idelalisib‐treated patients), fatal and/or severe diarrhea or colitis (14%–20%), fatal and/or serious pneumonitis (4%), fatal and/or serious infections (21%–48%), and fatal and serious intestinal perforation. Patients should be especially monitored for opportunistic infections such as CMV and Pneumocystis jirovecii. This safety profile has led to a reduced use of idelalisib in CLL, although the drug may be useful for controlling high‐risk disease [220].

#### 5.2.5 Combinations Using BTK Inhibitors and Anti‐CD20 Antibodies

**Ibrutinib and rituximab** combination therapy was shown to induce durable remissions in 40 patients with high‐risk CLL [221]. Treatment consisted of 28‐day cycles of once‐daily ibrutinib 420 mg together with rituximab (375 mg/m2, i.v., every week during cycle 1, then once per cycle until cycle 6), followed by continuous daily single‐agent ibrutinib 420 mg. Toxicity was mostly mild to moderate in severity. A long‐term follow‐up of the study [222] with a median duration of treatment of 41 months showed a response rate of 95%, with 23% complete remissions. Median PFS was 45 months, and 32 months in patients with a del(17p).

The HELIOS trial was a Phase 3 study with 578 patients with relapsed or refractory CLL/SLL. Patients received six courses of BR combined with either ibrutinib or placebo until disease progression or unacceptable toxicity [223]. PFS was significantly improved by the addition of ibrutinib to BR. IRC‐assessed PFS at 18 months was 79% in the ibrutinib group and 24% in the placebo group. The most frequent adverse events were neutropenia and nausea. Seventy‐seven percent of patients in the ibrutinib group and 74% in the placebo group reported Grade 3–4 events. Neutropenia and thrombocytopenia were the most common Grade 3–4 adverse events in both groups.

The ECOG‐ACRIN intergroup trial E1912 compared ibrutinib plus rituximab (IR) to fludarabine, cyclophosphamide, and rituximab (FCR) in 529 treatment‐naive CLL patients aged 70 or younger [224]. Patients were randomly assigned (2:1) to receive either IR for 6 cycles (after 1 cycle of ibrutinib alone) followed by continuous ibrutinib or 6 cycles of FCR. Results showed improved PFS for IR. With a median follow‐up of 5.8 years, IR outperformed FCR in both IGHV mutated and unmutated CLL [225]. Of the 354 patients assigned to IR, 214 (60.5%) remained on treatment. Discontinuations occurred due to disease progression (10.5%), adverse events (21.9%), or other reasons (6.8%). Progression was rare among those who stayed on ibrutinib, with a median time to progression after discontinuation of 25 months. Overall survival (OS) also favored IR [225]. In conclusion, IR therapy offered better PFS and OS than FCR, and continuous ibrutinib was well‐tolerated beyond 5 years in most patients.

**Ibrutinib and ofatumumab**. The combination of ibrutinib with ofatumumab was also investigated [226]. Overall, the study confirmed that the combinations of anti‐CD20 antibodies and ibrutinib are well tolerated, active therapeutic regimens. With the retraction ofatumumab from the market, these combinations are no longer used for the treatment of CLL.

**Ibrutinib and obinutuzumab**. The Illuminate study tested chlorambucil‐obinutuzumab against a combination of ibrutinib and obinutuzumab in elderly and comorbid patients [227]. This combination had shown promising results with uMRD responses in a Phase 2 trial [228]. The Illuminate study produced a significant PFS benefit for the combination of ibrutinib and obinutuzumab versus chlorambucil‐obinutuzumab. In the final analysis of this study with a median follow‐up of 45 months, ibrutinib plus obinutuzumab continued to show a sustained clinical benefit for PFS but not OS [229]. As the study did not contain an ibrutinib monotherapy arm, the benefit of adding obinutuzumab to ibrutinib remains unclear.

**Acalabrutinib plus obinutuzumab**. A Phase 1b/2 study generated a rationale for combining acalabrutinib and obinutuzumab [230]. Nineteen treatment‐naive and 26 relapsed/refractory patients with CLL were treated with acalabrutinib until progression and with obinutuzumab. Grade 3/4 adverse events occurred in 71% of patients. Overall response rates were 95% (treatment‐naive) and 92% (relapsed/refractory). Thirty‐two percent of treatment‐naive and 8% of relapsed/refractory patients achieved complete remission. At 36 months, 94% (treatment‐naive) and 88% (relapsed/refractory) were progression‐free.

The ELEVATE‐TN trial evaluated acalabrutinib alone or with obinutuzumab versus chlorambucil with obinutuzumab in 535 untreated CLL patients [231]. Acalabrutinib was given for 1 cycle before O to minimize infusion‐related reactions. Updated results showed longer median PFS for both acalabrutinib plus obinutuzumab and acalabrutinib (median not reached) compared to chlorambucil plus obinutuzumab (27.8 months), with hazard ratios of 0.14 and 0.23, respectively (*p* < 0.0001 for both) [232]. The estimated 72‐month PFS rates were 78%, 62%, and 17%. Median OS was not reached in any arm, but acalabrutinib plus obinutuzumab showed significantly longer OS than chlorambucil plus obinutuzumab (HR: 0.62; *p* = 0.0349). In high‐risk subgroups (unmutated IGHV and del(17p)/*TP53* mutations), PFS was also improved with acalabrutinib plus obinutuzumab and acalabrutinib compared to chlorambucil plus obinutuzumab. The overall response rate (ORR) was higher for acalabrutinib plus obinutuzumab (96%) and acalabrutinib (90%) compared to chlorambucil plus obinutuzumab (83%). Adverse events for acalabrutinib plus obinutuzumab included neutropenia (31%) and COVID‐19 (9%). Treatment continued in 54% of patients receiving acalabrutinib plus obinutuzumab and 47% for acalabrutinib, with discontinuations primarily due to adverse events. In conclusion, after a median follow‐up of 74.5 months, acalabrutinib plus obinutuzumab and acalabrutinib monotherapy showed sustained efficacy and safety, particularly in high‐risk patients, with significantly longer PFS for acalabrutinib plus obinutuzumab compared to acalabrutinib [232].

#### 5.2.6 Combinations Using VENETOCLAX or Other BCL2‐Antagonists

In a first attempt to introduce Bcl2‐antagonists into CLL therapies, oblimersen was tested in combination with fludarabine and cyclophosphamide in 241 patients with CLL [233, 234]. This combination achieved deep responses (CR/nPR) of 17% compared to 7% in the chemotherapy‐only group (*p* = 0.025). The study showed that the OS and the PFS were improved in patients who achieved at least a partial response. This study heralded the potential of combination therapies using Bcl‐2 antagonists.

##### 5.2.6.1 Venetoclax Plus CD20 Antibodies

A combination of **venetoclax and rituximab** was investigated in 49 patients with CLL with relapsed or refractory CLL or SLL and achieved encouraging results [235]. Overall, 42 (86%) of 49 patients achieved a response, including a complete response in 25 (51%) of 49 patients. Two‐year estimates for PFS and ongoing response were 82% and 89%, respectively. Negative marrow MRD was achieved in 20 (80%) of 25 complete responders and 28 (57%) of 49 patients overall.

In the Murano trial, 389 patients received venetoclax for up to 2 years (from day 1 of cycle 1) plus rituximab for the first 6 months (Ven‐R group) or bendamustine plus rituximab for 6 months (BR) [236]. At the 5‐year follow‐up, the median PFS was 53.6 months in the Ven‐R arm and 17 months in the BR arm, with a significant 5‐year‐OS advantage for Ven‐R (82.1% vs. 62.2%) [237]. The benefit was maintained across all clinical and biologic subgroups, including patients with del(17p). The rate of Grade 3 or 4 neutropenia was higher in the Ven‐R group than in the BR group, but the rates of Grade 3 or 4 febrile neutropenia and infections or infestations were lower with venetoclax than with bendamustine. These results established venetoclax plus rituximab as a new second‐line treatment in CLL.

**Venetoclax and obinutuzumab** were initially evaluated in 12 patients with previously untreated CLL and coexisting medical conditions as part of a run‐in phase of the CLL14 Phase 3 protocol and showed very encouraging results [238], in particular an overall response rate of 100% and no detectable (< 10^−4^) MRD in peripheral blood in 11 or 12 patients. The CLL14 protocol was a Phase 3 study comparing fixed‐duration treatment with venetoclax and obinutuzumab (Ven‐Obi) to chlorambucil and obinutuzumab (Clb‐Obi) in untreated CLL patients with coexisting conditions [74, 239]. A total of 432 patients were randomized equally between the two groups. Grade 3 or 4 neutropenia occurred in 52.8% of the Ven‐Obi group and 48.1% of the Clb‐Obi group, while Grade 3 or 4 infections occurred in 17.5% and 15.0%, respectively. The Ven‐Obi group achieved a uMRD remission rate of 76%. Updated findings after a median follow‐up of 76.4 months showed superior PFS for Ven‐Obi (median 76.2 months) compared to Clb‐Obi (36.4 months), with benefits seen in patients with *TP53* alterations or unmutated IGHV genes [75]. The 6‐year OS rate was 78.7% for Ven‐Obi versus 69.2% for Clb‐Obi. In the Ven‐Obi arm, the presence of del(17p), unmutated Light genes, and a lymph node size of ≥ 5 cm were independent prognostic factors for shorter PFS.

The CLL13/GAIA trial of an international cooperative group, led by the GCLLSG, compared the efficacy of four therapies in untreated, fit patients with CLL without del(17p) or *TP53* mutation [73]. It evaluated standard chemoimmunotherapy (FCR or BR), venetoclax and rituximab (Ven‐R), obinutuzumab and venetoclax (Ven‐Obi), and venetoclax, obinutuzumab, and ibrutinib (Ven‐Obi‐Ibr). The primary endpoints were the rate of uMRD at month 15 and PFS, with Ven‐Obi‐Ibr showing a 3‐year PFS of 87.7%, Ven‐Obi at 90.5%, and standard therapy at 75.5%. At month 15, MRD negativity was significantly higher in the Ven‐Obi (86.5%) and Ven‐Obi‐Ibr (92.2%) groups compared to standard therapy (52.0%) and Ven‐R groups (57.0%). A follow‐up with a median of 50.7 months showed that patients in the Ven‐Obi group had significantly longer PFS than those in the chemoimmunotherapy and Ven‐R groups [240]. The estimated 4‐year PFS rates were highest in the Ven‐Obi‐Ibr group (85.5%), followed by Ven‐Obi (81.8%), and lower for the other groups. Neutropenia was the most common severe treatment‐related adverse event, with a few treatment‐related deaths, particularly in the Ven‐Obi‐Ibr group.

Overall, the CLL13 and CLL14 trials support the use of 1‐year fixed‐duration Ven‐Obi in previously untreated CLL patients. These fixed‐duration regimens offer a more effective and less toxic first‐line therapy for patients with CLL, with sustained long‐term survival and quality of life benefits.

##### 5.2.6.2 Combinations Using Venetoclax and BTK Inhibitors

The combination of venetoclax, a BCL‐2 inhibitor, with BTK inhibitors such as ibrutinib, acalabrutinib, pirtobrutinib, and zanubrutinib, has shown promising results in the treatment of chronic lymphocytic leukemia (CLL) to maximize therapeutic efficacy, overcome resistance mechanisms, and improve patient outcomes.

###### 5.2.6.2.1 Venetoclax and Ibrutinib

The CLARITY trial tested a combination of ibrutinib with venetoclax in patients with relapsed or refractory CLL [241]. The primary endpoint was eradication of MRD after 12 months of combined therapy. After 12 months of ibrutinib plus venetoclax, MRD negativity was achieved in the blood of 28 out of 53 (53%) and the marrow of 19 patients (36%). Forty‐seven patients (89%) responded, and 27 (51%) achieved a complete remission. After a median follow‐up of 21.1 months, one patient progressed, and all patients were alive. A single case of biochemical tumor lysis syndrome was observed. Other adverse effects were mild or manageable and most commonly were neutropenia or GI events.

Another Phase 2 study investigated ibrutinib and venetoclax in 80 previously untreated high‐risk, older patients with CLL [242, 243]. All patients had at least one of the following features: del(17p), mutated *TP53*, del(11q), unmutated *IGHV*, or an age of 65 years or older. Patients received ibrutinib monotherapy for 3 cycles, followed by the addition of venetoclax. Combined therapy was administered for 24 cycles. At a median follow‐up time of 38.5 months [243], the combination therapy with ibrutinib and venetoclax showed promising results, with durable remissions and high activity seen across high‐risk disease subgroups. The 3‐year PFS was 93%, and the 3‐year OS was 96%.

The GLOW trial compared ibrutinib and venetoclax to chlorambucil and obinutuzumab in previously untreated CLL/SLL [244, 245]. The study enrolled patients aged ≥ 65 years or 18–64 years with cumulative illness rating scale (CIRS) score > 6 or creatinine clearance < 70 mL/min. One hundred and six patients received 3 cycles of ibrutinib followed by 12 cycles of ibrutinib plus venetoclax and 105 patients received 6 cycles of standard dose chlorambucil plus obinutuzumab. The median age was 71.0 years (34.1%; ≥ 75 years). At a median of 46 months of follow‐up [245], PFS was superior for the ibrutinib‐venetoclax group, with 42‐month PFS rates were 74.6% for ibrutinib‐venetoclax and 24.8% for chlorambucil‐obinutuzumab. There were 15 deaths in the ibrutinib‐venetoclax group (of which three were due to post‐treatment infections) and 30 deaths in the chlorambucil‐obinutuzumab group (with 10 attributed to post‐treatment infections).

The FLAIR trial compared the combination of venetoclax and ibrutinib to the traditional chemoimmunotherapy regimen of fludarabine, cyclophosphamide, and rituximab (FCR) [76]. At a median follow‐up of 43.7 months, disease progression or death occurred in 12 patients in the venetoclax‐ibrutinib group versus 75 in the FCR group. The combination also showed a significant improvement in OS. Death occurred in 9 patients in the venetoclax‐ibrutinib group compared to 25 in the FCR group. After 5 years, 65.9% of patients had uMRD in the bone marrow, and 92.7% had uMRD in the peripheral blood [76]. The combination was generally well‐tolerated, with no unexpected side effects. The risk of infection was similar in both groups. The percentage of patients with cardiac serious adverse events was higher in the ibrutinib‐venetoclax group than in the FCR group (10.7% vs. 0.4%). The FLAIR trial used a flexible treatment duration, which was determined by the time point at which an undetectable MRD was achieved. Patients with persistent detectable MRD continued treatment, for a maximum of up to 6 years. The duration of ibrutinib–venetoclax therapy was determined according to the MRD‐directed approach, with 146 of 260 patients stopping treatment owing to MRD stopping rules after 24–60 months of ibrutinib–venetoclax treatment. Kaplan–Meier estimates of the percentage of patients who had stopped treatment by specific time points were by 24 months, 28.9%; by 36 months, 58.0%; and by 60 months, 78.4%. Five patients restarted ibrutinib–venetoclax and were alive and progression‐free at the last follow‐up. In summary, the results suggest that a time‐limited, MRD‐guided application of venetoclax and ibrutinib could be a more effective and safer alternative to traditional chemoimmunotherapy for CLL.

###### 5.2.6.2.2 Venetoclax and Acalabrutinib

The Phase 2 CLL2‐BAAG trial investigated an MRD‐guided combination of acalabrutinib, venetoclax, and obinutuzumab (after optional bendamustine debulking) in 45 patients with relapsed/refractory CLL [246]. With a median observation time of 36.3 months and all patients off‐treatment for a median of 21.9 months, uMRD < 10–4 in PB was achieved in 42 of the 45 patients (93.3%), including 17 of 18 (94.4%) previously exposed to venetoclax/BTKi and 13 of 14 (92.9%) with *TP53* aberrations. The estimated 3‐year progression‐free and OS rates were 85.0% and 93.8%, respectively.

The AMPLIFY trial investigated the use of acalabrutinib in combination with Ven‐Obi. The study investigated 867 patients treated with venetoclax‐acalabrutinib (Ven‐Aca, *n* = 291) or Ven‐Obi plus acalabrutinib (Ven‐Obi‐Aca, *n* = 286) or chemoimmunotherapy with FCR/BR (*n* = 290) with a median age 61 years [247]. At a median follow‐up of 41 months, both Ven‐Aca and Ven‐Obi‐Aca showed an improvement in PFS over chemoimmunotherapy.

###### 5.2.6.2.3 Venetoclax and Pirtobrutinib

In a Phase 1b trial, patients with relapsed or refractory chronic lymphocytic leukemia (CLL) were treated with fixed‐duration pirtobrutinib plus venetoclax (PV) or pirtobrutinib plus venetoclax and rituximab (PVR) [248]. Overall response rates were 93.3% for PV and 100% for PVR, with high rates of minimal residual disease negativity. The combination was well tolerated and showed promising efficacy, especially in patients previously treated with covalent BTK inhibitors. Further studies are needed to confirm these findings.

###### 5.2.6.2.4 Venetoclax and Zanubrutinib

A multicenter, Phase 2 study tested a combination of zanubrutinib, obinutuzumab, and venetoclax in 39 patients (median age 62 years) with treatment naïve CLL/SLL [249]. Thirty‐nine patients had unmutated IGHV, and five (13%) had del(17p) or *TP53* mutation. The primary endpoint was the proportion of patients reaching uMRD in both the peripheral blood and bone marrow. After a median follow‐up of 25.8 months, 89% had uMRD in the blood and marrow. Overall, this combination showed good efficacy and reasonable safety.

###### 5.2.6.2.5 Triple Drug Combinations

The CLL2‐GIVe trial showed very encouraging results for the triple combination of obinutuzumab, ibrutinib, and venetoclax in previously untreated patients with high‐risk CLL [250]. The complete remission rate was 58.5%, and the 36‐month PFS was 79.9%. Adverse events included neutropenia, infections, and cardiovascular toxicity, with most events occurring during induction and decreasing over time.

Davids et al. explored an MRD‐guided triplet therapy with acalabrutinib, venetoclax, and obinutuzumab in a Phase 2 study in 37 treatment naïve patients with CLL (median age 63). Treatment involved 28‐day cycles of acalabrutinib followed by combining it with obinutuzumab and escalating venetoclax doses. Patients could discontinue therapy if they achieved uMRD. At cycle 16 day 1, 38% of participants achieved complete remission with uMRD. The most common severe adverse effect was neutropenia (43%), and no deaths occurred. In summary, the triple drug combinations show a manageable safety profile and hold promise as a treatment for high‐risk patients.

Combining venetoclax with BTK inhibitors like ibrutinib, acalabrutinib, pirtobrutinib, and zanubrutinib has demonstrated significant potential in treating CLL. However, we still need long‐term data from ongoing clinical trials to determine the impact on OS and to optimize treatment plans. Additionally, using MRD‐driven treatment approaches may help tailor therapy to individual patient needs, potentially leading to better outcomes and shorter treatment durations. Future research will continue to optimize treatment plans, manage side effects, and explore combination therapies with other new drugs.

#### 5.2.7 Combinations of Checkpoint Inhibitors With BCL2 or BTK Inhibitors

Richter transformation (RT) is defined as the development of an aggressive lymphoma that is associated with a very poor response to chemotherapy and short survival. Two recent Phase 2 protocols have generated interesting data using a combination of checkpoint inhibitors with BTK inhibitors. In one study patients with RT received a combination of the PD‐1 inhibitor tislelizumab plus the BTK inhibitor zanubrutinib for 12 cycles [251]. Patients responding to treatment received a maintenance treatment with both agents. Of 59 enrolled patients, 48 received at least two treatment cycles and comprised the analysis population. Ten patients (20.8%) had received previous RT‐directed therapy. Twenty‐eight out of 48 patients responded to induction therapy with an overall response rate of 58.3%, including 9 (18.8%) complete and 19 (39.6%) partial responses. The median PFS was 10.0 months. The 12‐month OS rate was 74.7%. The most common adverse events were infections (18.0%), gastrointestinal disorders (13.0%), and hematological toxicities (11.4%). The most interesting observation of the trials was that some patients showed long‐lasting responses over several months to years.

In a similar protocol by an Italian and Swiss consortium, the PD‐L1 inhibitor atezolizumab was used in combination with venetoclax and obinutuzumab in patients with DLBCL‐RT. In this trial, patients had not previously received treatment for DLBCL‐RT [252]. No previous treatment with any of the drugs in the triplet combination was allowed. Patients received 35 cycles of 21 days of intravenous obinutuzumab and intravenous atezolizumab combined with continuous oral venetoclax. The primary endpoint was the overall response rate at day 21 of cycle 6 in the intention‐to‐treat population. Twenty‐eight patients were enrolled. Nineteen of 28 patients showed a response, yielding an overall response rate of 67.9%. Treatment‐emergent adverse events that were Grade 3 or worse were reported in 17 of 28 patients, with neutropenia being the most frequent (39%). Serious treatment‐emergent included infections (18%), with two (7%) deaths attributable to adverse events (one from sepsis and one from fungal pneumonia), which were not considered as directly treatment‐related by the investigators. Six (21.4%) patients had immune‐related adverse events, none of which led to discontinuation. No tumor lysis syndrome was observed.

Together, both studies illustrate the potential of using blockade of PD‐1 in combination with targeted agents for the treatment of Richter transformation.

### 5.3 Novel Therapeutic Modalities for Patients Refractory or Resistant to BCL2 Inhibitors and BTK Inhibitors (Double Refractory CLL)

Treatment with BTK and BCL2 inhibitors (BTKi; BCL2i) has replaced chemoimmunotherapy as first‐line therapy in most CLL patients [73, 132, 239]. While continuous BTKi monotherapy effectively controls disease, MRD remains detectable [253], and most patients either relapse or need subsequent therapy due to adverse events. In contrast, fixed‐duration combination regimens achieve undetectable uMRD [76, 254], but the disease recurs in a relevant fraction of patients. In this situation, switching from BTKi to BCL2i or vice versa is a current standard of care. When patients become refractory to both classes of agents (BTKi and BCL2i), a condition which has been termed “double refractory” (2R) [39], no standard therapy exists and novel modalities are currently being explored.

**FIGURE 2:**
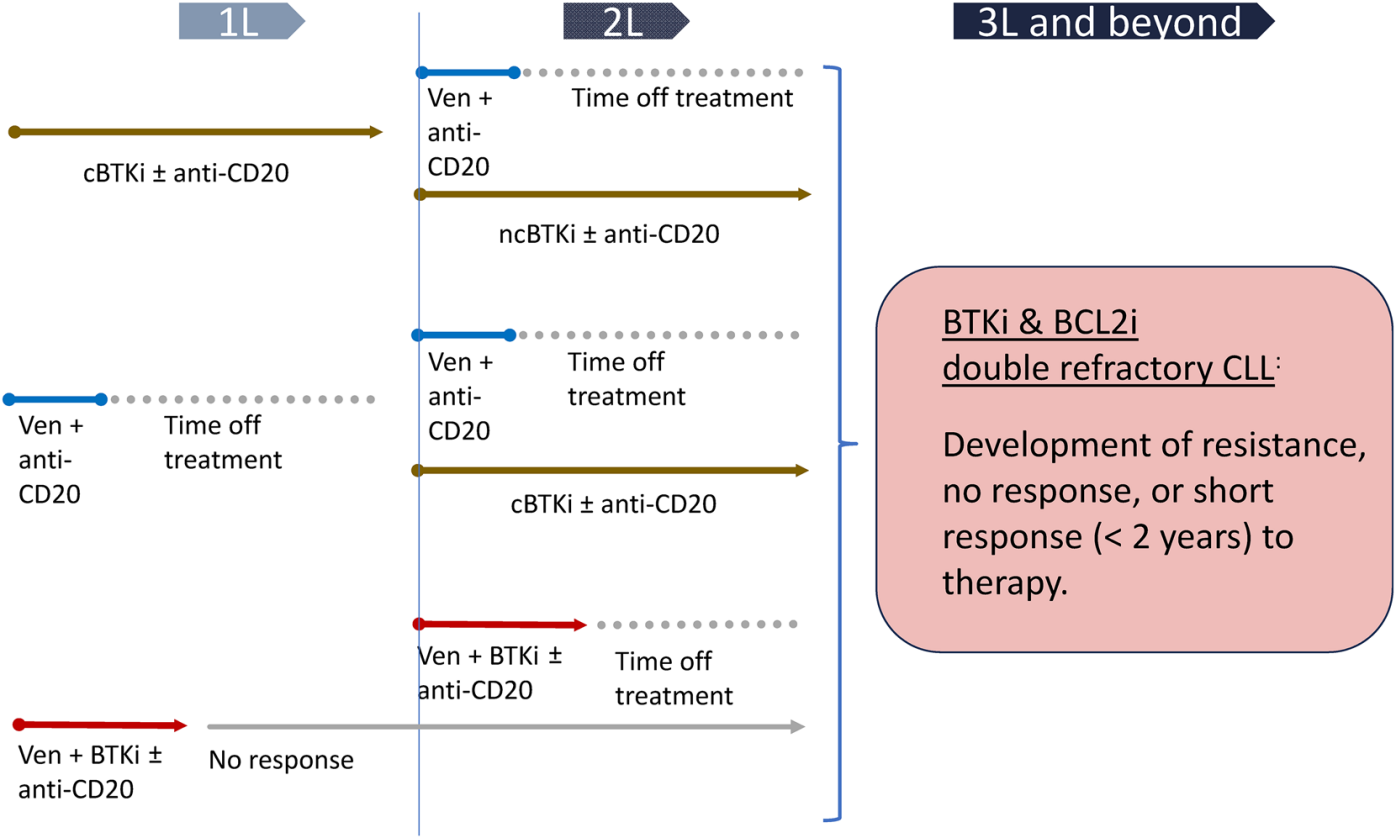
Treatment sequences leading to CLL being refractory to both BCL2 inhibitors and BTK inhibitors, called double refractory CLL (2R CLL).

The following conditions can lead to 2R CLL (Figure 2):

1. CLL patients who are treated first‐line with continuous BTKi monotherapy and receive time‐limited venetoclax combinations at relapse and then become resistant to venetoclax as well.
2. Fixed‐duration BCL2i combinations, especially with CD20 antibodies, can lead to long‐lasting remissions with sustained uMRD. Some patients may relapse after first‐line therapy [254, 255, 256]. Those with prolonged remission may be retreated with the same or similar BCL2 inhibitor combinations, while patients with early relapses or those on continuous therapy are switched to BTK inhibitors as second‐line treatment. Though these therapies are often effective, some patients may relapse again, becoming “double refractory” (2R) [257].
3. Time‐limited combinations of BCL2i and BTKi show promise as first‐line treatments with long‐lasting disease control [73, 76, 258, 259]. Upon relapse, patients will have been exposed to both therapies [250]. More studies are needed to identify which patients can be retreated with these regimens versus those needing novel second‐line therapies. A treatment‐free remission of over 3–4 years may indicate eligibility for retreatment, while shorter remissions may require experimental approaches.

For 2R CLL patients, a widely accepted treatment standard is lacking [257]. With the rise of BTKi and BCL2i use, the number of 2R patients is expected to increase, especially following the recent approval of the ibrutinib and venetoclax combination as first‐line treatment in Europe [258, 259]. This group often has high‐risk genetics and an elevated risk of Richter's transformation. The median OS in heavily pretreated 2R‐CLL is only 8–27 months [260, 261, 262], with limited benefits from retreatment using venetoclax plus ibrutinib, lasting 7.5–9.3 months before disease progression [261, 263]. Future 2R patients may fare better with less pretreatment, but a significant medical need remains. In addition, many patients will require alternatives due to side effects from BTKi or BCL2i [147]; some patients may switch to a new generation of BTKi that offers comparable disease control with fewer cardiovascular side effects [145, 147, 150, 264].

Taken together, 2R CLL patients represent a new and major medical need requiring the development of novel treatment strategies.

#### 5.3.1 Approved Therapeutic Modalities for 2R CLL

**Chemoimmunotherapy**. Data on the use of CIT in 2R patients is sparse. As they commonly carry genetic *TP53* dysfunction, the effectiveness of CIT is limited [11, 198]. Moreover, a large proportion of 2R CLL patients is old or frail, excluding the use of intense CIT regimens or allogeneic SCT.

**PI3K inhibitors**. There is limited knowledge regarding the use of PI3Ki in 2R CLL. One study reported an ORR to PI3KI of 47% and a median PFS of only 5 months in 17 double‐exposed patients [265]. Other trials in relapsed/refractory (R/R) CLL demonstrated high response rates to PI3Ki, including patients with high‐risk genetics, suggesting efficacy in 2R patients [119, 216]. However, immune‐mediated side effects including Grade 3 diarrhea, colitis, and pneumonitis remain a concern [119, 216, 266].

**Non‐covalent BTKi (pirtobrutinib)**. Upon treatment with covalent BTKi (ibrutinib/acalabrutinib/zanubrutinib), resistance mutations may drive relapses (e.g., detected in 87% of patients who progressed on ibrutinib [267]). Most commonly these occur within the inhibitor binding site at cysteine residue 481 (C481) and reduce binding and efficacy of cBTKi, while in other patients additional or single mutations of the downstream signaling protein PLCγ2 were identified as cause for BTKi resistance [134, 267].

Pirtobrutinib has demonstrated good efficacy in cBTKi‐pretreated 2R patients (ORR 70%; median PFS 16.8 months) with a favorable toxic‐effect profile and retains its effectiveness in mutated BTK‐C481 [268]. Notwithstanding, PLCγ2‐mutated patients exhibited reduced response rates [268] and new resistance mutations against pirtobrutinib have been described [269].

#### 5.3.2 Novel, Experimental Agents for 2R CLL

**BTK degraders**. As explained above, these drugs induce the degradation of the target via ubiquitylation, offering a novel mechanism to overcome BTK resistance mutations. Several of these compounds effectively degrade BTK in vitro, including those with C481 mutations [153], and are currently evaluated in clinical trials in highly pretreated CLL patients [270]. Their specific use in 2R patients remains to be evaluated.

**BCL2 and MCL1 inhibitors**. Besides mutations in the BCL2 gene (e.g., G101V), which reduce binding and effectiveness of venetoclax, a plethora of further mechanisms counteract BCL2i [271]. Foremost, the deregulation of apoptosis‐regulating proteins resulting from leukemia‐intrinsic (e.g., genetic mutations, amplification, or epigenetic regulation [271, 272]) and/or processes driven by the tumor microenvironment promote resistance to BCL2i [273, 274, 275]. Therefore, new BCL2i and inhibitors targeting alternative anti‐apoptotic proteins, such as MCL1 (MCL1i) and Bcl‐XL (BclXLi) are being evaluated, and some may show activity to overcome venetoclax resistance [276]. Moreover, targeting other antiapoptotic proteins has shown severe side effects (MCL1i: hematotoxicity, cardiotoxicity, intestinal and liver toxicity, BclXLi: thrombocytopenia), which may limit their use in 2R CLL patients [277, 278].

**Agents targeting ROR1**. In contrast to healthy B cells, CLL cells mostly express the surface receptor ROR1 [279]. ROR1 signaling, induced by WNT5a, activates leukemic cells and high expression levels are associated with lymphomagenesis, dismal outcome, and venetoclax resistance [279, 280, 281]. Zilovertamab, a mROR1Ab disrupts ROR1 signaling and inhibits the growth of BCL2i resistant cells [280]. Therefore, ROR1 offers a promising target for antibodies, BITEs and CAR‐T cells, and demonstrated promising efficacy in combination with ibrutinib [282]. Similarly, early clinical data evaluating a ROR1‐BITE showed promising preliminary results [283].

**Bispecific antibodies (BsAbs) and T cell engagers (BITEs)** bind two different antigens, mostly linking T cells to a target. These agents are currently approved for relapsed hematological malignancies [284, 285]. Preliminary data indicates good efficacy, induction of T cell proliferation, activation and leukemia clearance in vitro [286], and early clinical results of epcoritamab (CD3xCD20) show deep responses with 53% ORR in double‐exposed CLL [287]. Since some BsAbs showed improved effectiveness in combination with BTKi/BCL2i in vitro [286, 288], combining BsAbs with BTKi/BCL2i and/or ICi appears a promising strategy to boost T cell function.

**CAR‐T cells** represent another option for treating 2R CLL (see above). However, in the context of refractory CLL cases, CR rates and long‐term responses to CD19 CAR‐T cells were lower than in other lymphomas [289, 290]. Explanations for this phenomenon include impaired formation of the immune synapse, the production of extracellular vesicles attenuating CAR‐T cell function [290], and metabolic perturbation of CAR‐T cells [291, 292, 293]. Importantly, the efficacy of CAR‐T cell products is also reduced by production from a largely exhausted or dysfunctional T cell pool. Therefore, strategies are needed to improve T cell fitness before harvesting, during manufacturing, and after transfusion. Methodologies to modulate the tumor microenvironment appear particularly attractive in this regard. Accordingly, ibrutinib was shown to restore T cell function, facilitate production, reduce CRS rates, and increase the in vivo efficacy of CAR‐T cells in CLL [294, 295, 296, 297].

#### 5.3.3 Combination Therapies to Rewire and Modulate the TME for the Treatment of 2R CLL

We have proposed a comprehensive strategy for treating 2R CLL that utilizes the cellular and molecular elements of the leukemic microenvironment (TME). Given the complexity of the TME, effective therapies need to target multiple interactions. Monotherapies have shown limited success, particularly in R/R patients. Combining potent anti‐neoplastic agents with TME‐rewiring therapeutics may enhance treatment efficacy and restore an anti‐tumor environment, thus preventing resistance. When designing study concepts, it is essential to anticipate both synergistic and opposing effects of drugs on the TME. Potential outcomes include (a) creating synergistic effects through shared mechanisms, (b) preparing the TME for interventions, (c) overcoming resistance, and (d) adding anti‐leukemic efficacy. Agents with direct activity on both the leukemic cells and on the TME (like BTKi, PI3Ki, dasatinib, and lenalidomide) are particularly promising combination partners in this context.

The TME dynamically adapts to therapies, while the initial composition and polarization also critically govern treatment outcomes. In this regard, the efficacy of immunotherapies depends on the immune cell landscape at treatment initiation as well as treatment‐associated shifts in composition. The presence of active T cells may augment the effectiveness of CAR‐T cells and immune checkpoint inhibitors [298], while exhausted T cells and T_regs_ can prevent the success of such treatments, including BsAb [299]. Tumor‐associated macrophages and cancer‐associated fibroblasts may also negatively impact CAR‐T cell activity [298, 300]. For example, a successful modulation of the TME composition in CLL has been demonstrated in vitro for epcoritamab by pretreatment with BTKi [288]. Moreover, a reduction of the leukemic burden may shift the effector: target ratio and improve the efficacy of CAR‐T cells, ICi, and BsAbs [301].

As **sequential use** of cytotoxic and/or TME modulating agents may prepare the TME for more effective immunotherapy, we have proposed a strategy for TME‐directed therapies that will be tested in clinical trials for 2R patients. These therapies will be performed in sequential steps and use combinations of kinase inhibitors (dasatinib) and T‐cell engaging agents (bispecific antibodies) [39].

## 6 Selecting the Right Treatment: How to Treat CLL?

### 6.1 Parameters to Be Considered

Given the impressive number of choices, the selection of the optimal treatment for a given CLL patient has become a task that requires experience, good clinical judgment, and an appropriate use of diagnostic tools.

In addition to leukemia‐related parameters, the newer agents may induce a number of specific side effects. Therefore, the pre‐existing comorbidities (e.g., cardiomyopathies, arrhythmia, renal failure), the comedication (e.g., CYP inhibitors, anticoagulants), and also the individual preference (time‐limited vs. indefinite treatment), and finally even economic considerations need to be discussed with the patient before the initiation of treatment.

Despite its efficacy and widespread use, indefinite BTKi monotherapy of patients with CLL comes with some drawbacks: an increased financial burden, relatively high rates of cardiovascular side effects as well as resistance mutations and relapses after drug discontinuation [302, 303, 304]. Therefore it appears advantageous to use fixed‐duration combination therapies with venetoclax, BTK inhibitors, and/or obinutuzumab that aim to achieve uMRD, durable responses that allow to create treatment free time for the patient and have shown to be safe and tolerable.

The following parameters need to be considered before recommending a treatment for CLL:

1. The clinical stage of the disease
2. The symptoms of the patient.
3. The fitness and concomitant diseases of the patient, particular with regard to the specific organ toxicity of the newer, targeted agents.
4. The genetic risk of leukemia.
5. The treatment situation (first vs. second line, response vs. non‐response to the last treatment).

Using these five parameters, the following recommendations can be given:

### 6.2 First Line Treatment

In a patient with advanced (Binet C, Rai III‐IV) or active, symptomatic disease (Table 2), treatment should be initiated. As the novel agents are less toxic when compared to chemoimmunotherapies, the fitness of the patients no longer plays a major role in the treatment decision. Recent evaluations of the GCLLSG across the trials CLL13 and CLL14 have shown that the efficacy and toxicity of venetoclax‐obinutuzumab do not depend on the fitness of the patients (as classified according to the former go‐go and slow‐go categories) [305]. Therefore, patients in need of treatment should be offered a fixed‐duration therapy with venetoclax plus obinutuzumab, a monotherapy with a second‐generation BTK inhibitor (acalabrutinib, zanubrutinib), or a combination of venetoclax with a BTK inhibitor regardless of their fitness. No clear survival benefit has been documented for any of these options. The potential side effects of the different modalities need to be assessed by careful cardiovascular examination and monitoring when using BTK inhibitors, or by adapted measures to prevent tumor lysis or kidney failure in patients treated with venetoclax combinations. These different consequences should be discussed with the patient.

**FIGURE 3:**
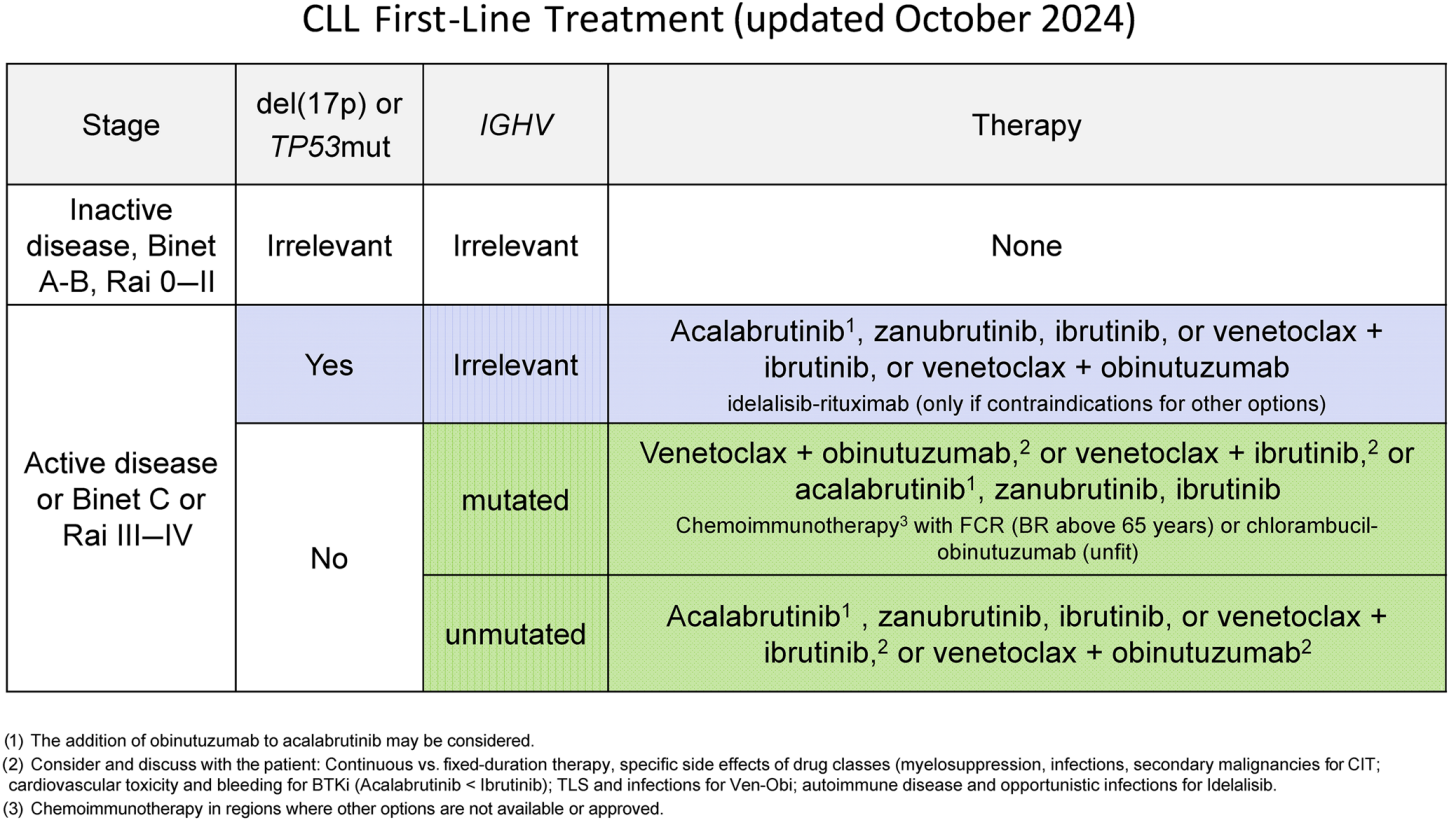
Updated treatment algorithm for patients with CLL in first‐line indications.

Patients with del(17p) or *TP53* mutations represent a somewhat separate category. In these patients, chemoimmunotherapy should be avoided. It is recommended that these patients receive a BTK inhibitor alone or in combination with venetoclax, as these agents have shown good long‐term control of this condition [129] (Figure 3). In these patients, an allogeneic stem cell transplantation may be discussed at the second relapse [306].

For some patients in good physical condition [307], chemoimmunotherapy with FCR can still be debated in countries, where some of the targeted agents are not available, to achieve long‐term remissions or cures in patients with a mutated IGVH gene and without genetic p53 dysfunction (Figure 3).

### 6.3 Second Line Treatment

**FIGURE 4:**
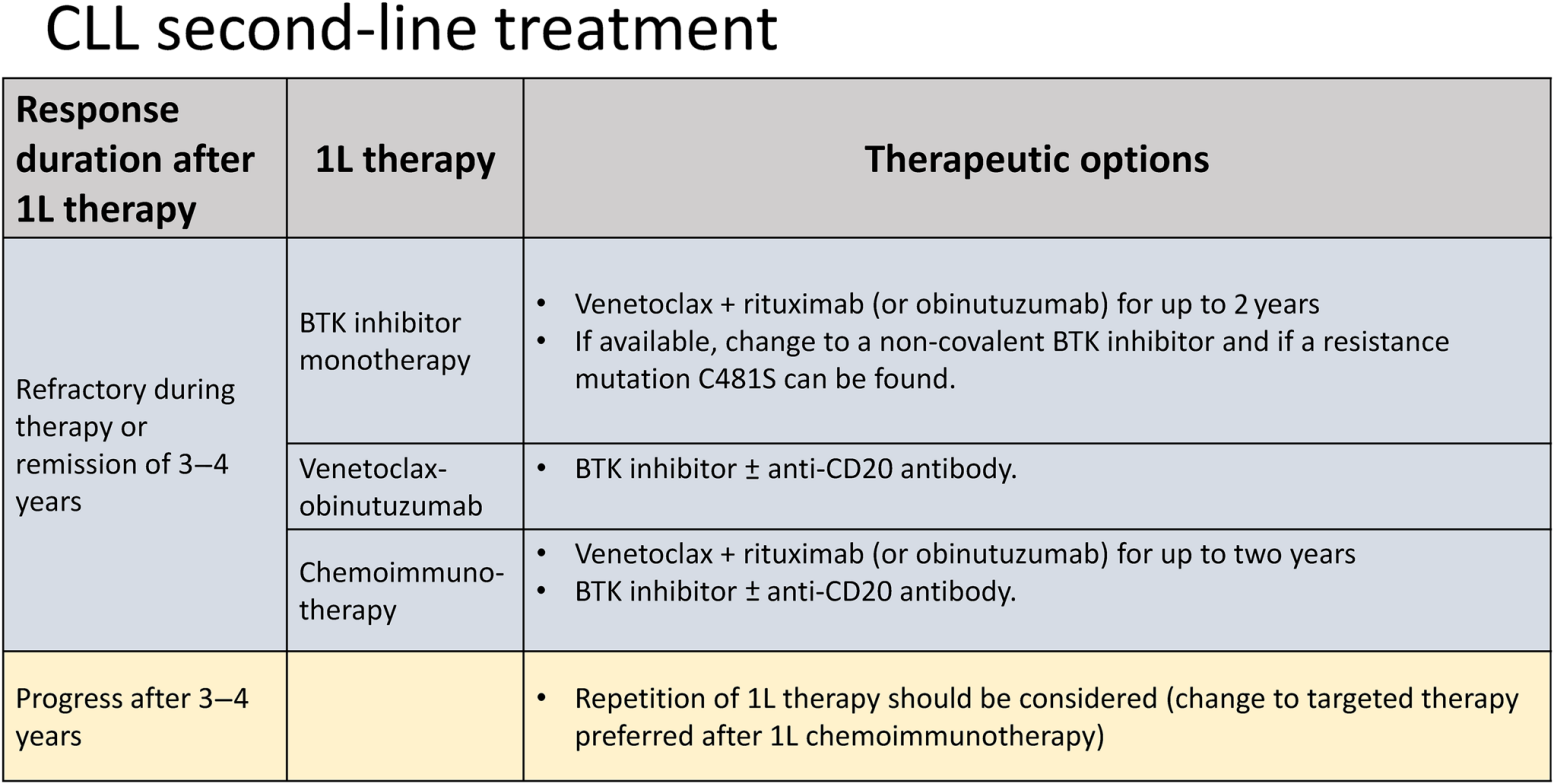
Updated treatment algorithm for patients with CLL in second‐line indications.

Figure 4 summarizes the principles of managing of patients at relapse according to the duration of remission and physical fitness. As a general rule, the first‐line therapy may be repeated if the duration of the first remission exceeds 3–4 years. With novel therapies, the point to repeat a given therapy may have shifted to 4 years, although no evidence exists for this recommendation.

The choice is different in treatment‐refractory CLL (as defined by an early relapse within 3–4 years after the first treatment), and in relapsed cases with a chromosomal aberration del(17p). In these cases, the second regimen should be different from the first and a potent second‐line regimen should be selected.

The following options exist:

1. For patients with a BTK inhibitor as a first‐line therapy:
  - Venetoclax in combination with rituximab (or obinutuzumab) for up to 2 years.
  - Changing to a non‐covalent BTK inhibitor if available and if a resistance mutation C481S can be found.
2. For patients with a venetoclax‐obinutuzumab combination therapy:
  - Repeat if initial remission lasted for more than 3–4 years.
  - Use a BTK inhibitor with or without an anti‐CD20 antibody.
3. For patients who have received a chemoimmunotherapy as first‐line therapy:
  - Use a BTK inhibitor with or without an anti‐CD20 antibody.
  - Venetoclax in combination with rituximab (or obinutuzumab) for up to 2 years (MURANO).

### 6.4 Third Line Therapies

As discussed above, the situation of double‐refractoriness (2R) (Figure 2) is a new medical need and leaves a limited number of treatment options.

1. PI3K inhibitors (idelalisib and rituximab);
2. Cellular therapies such as CART cell therapy [294] or allogeneic stem cell transplantation with curative intent [306];
3. Use of chemoimmunotherapy or alemtuzumab, although with limited success [107, 308].

These patients have a high risk for poor outcomes and therefore represent good candidates for inclusion in experimental protocols and the test of novel agents (see above). It should be mentioned that in some patients, the re‐use of a previous, second‐to‐last therapy can be discussed after the failure of a given therapeutic modality, as resistance‐defining mutations for this second‐to‐last treatment may have disappeared.

### 6.5 Current Challenges and Uncertainties

As novel agents have emerged for the treatment of CLL, the **optimal sequencing and combination strategies** remain to be established for these agents. So‐called “real‐world” observations suggest that ibrutinib appears superior to idelalisib when used as the first kinase inhibitor [309]. In the setting of ibrutinib failure, venetoclax therapy appears superior to both idelalisib and chemoimmunotherapy [309, 310], while patients refractory to venetoclax showed the best outcomes when consequently treated with ibrutinib [311, 312]. These data are largely derived from registries or retrospective cohort studies, lending support for randomized studies that test different sequencing strategies.

The sequenced application of single agents rarely leads to **uMRD responses**. In contrast, their combined application may induce deep and durable remissions with long‐treatment‐free intervals. One of these trial concepts uses sequential, targeted therapies to eradicate residual disease [82, 313]. Moreover, combinations of all available drugs, as well as novel strategies to prevent the clonal evolution of CLL, need to be investigated [314, 315] to achieve long‐lasting remissions or even cures for patients with CLL. So far, results obtained by these combination therapies appear promising, in particular when combining anti‐CD20 antibodies with targeted agents [226, 228, 235, 236, 238, 241, 242, 316, 317]. While ibrutinib has been tested in combination with anti‐CD20 antibodies and yielded high response rates, the choice of the antibody has an impact on the efficacy. The time‐limited combination treatments of ibrutinib and obinutuzumab showed an MRD‐negativity rate of 48%, while ibrutinib and ofatumumab only yielded 14%. The CLL2‐BAG protocol (bendamustine, venetoclax, and obinutuzumab) yielded excellent overall response and uMRD response rates of around 90% both in treatment naïve and pre‐treated patients [318]. Similarly, the Murano trial produced uMRD responses in 64% of the 130 patients who completed the 24‐month venetoclax plus rituximab treatment, translating into significantly longer PFS [236]. Most importantly, these studies demonstrated that the majority of uMRD remissions were sustained for more than 1 year after the end of the study treatment [318, 319]. Venetoclax and ibrutinib also appear to achieve deep remissions. Two Phase 2 studies evaluating the use of this combination have been described above [241, 242]. Another trial combined the three most promising, approved agents (obinutuzumab, ibrutinib, and venetoclax), yielding a rate of uMRD responses of 67% in the treatment‐naïve cohort of the study [320].

The biologically informed combination of targeted agents has paved the way for the development of regimens that induce **deep**, **uMRD remissions with the possibility of discontinuing therapy**. This limited‐duration treatment concept is different from continuous targeted therapies, in particular with BTK inhibitors, that rarely induce uMRD remissions but achieve substantial disease control. It is so far unclear which of the two paradigms creates the greatest benefit for patients with CLL or specific subgroups, for example, patients with high‐risk disease. The ongoing CLL17 study of the GCLLSG (NCT04608318) addresses this very important question by randomizing patients with previously untreated CLL to either ibrutinib continuous monotherapy, fixed‐duration venetoclax‐obinutuzumab or fixed‐duration venetoclax‐ibrutinib.

When comparing different trials for **relapsed patients with CLL**, it becomes evident that all combinations using targeted agents (idelalisib, venetoclax, obinutuzumab, ibrutinib) are more potent than chemoimmunotherapy concerning key variables of efficacy such as overall response rate, complete remissions, uMRD remissions, PFS, and OS. These results justify the broad use of targeted agents, alone or in combination for second‐line therapy of CLL. In contrast, combining chemoimmunotherapy such as BR with ibrutinib or idelalisib has not yielded satisfactory benefits.

Another use of kinase inhibitors may allow to **enhance the function of T cells** [321]. It was shown that ≥ 5 cycles of ibrutinib therapy improved the expansion of CD19‐directed CAR T cells (CTL019), in association with a decreased expression of the immunosuppressive molecule programmed cell death 1 on T cells and of CD200 on B‐CLL cells [297]. Two clinical studies recently showed that this effect can be translated into higher efficacy of CAR‐T cells when combined with ibrutinib, yielding high response rates and a trend toward deeper remissions compared to CAR‐T cell infusions alone [322, 323].

Finally, despite the tremendous progress in our understanding and treatment of CLL, new challenges are emerging. As the majority of patients treated with targeted agents are not cured, disease relapses will eventually occur after exposure to BTK, PI3K, or BCL2 inhibitors. In particular, salvage options for disease that is **refractory to BTK and BCL2 inhibitors** are limited, and the outcome of patients with double‐refractory disease is quite poor [324]. For this group of patients, alternative therapeutic concepts are needed (see above).

In any case, the management of CLL will continue to undergo a very dynamic development. Therefore, we must continue to work toward the long‐term control of this disease by including our patients in current clinical trials. Moreover, in such a fast‐developing era of medicine bi‐annually updated recommendations offer the possibility to constantly monitor and summarize the clinically relevant progress in chronic lymphocytic leukemia management.

## Conflicts of Interest

The author declares institutional research support by Abbvie, AstraZeneca, Beigene, Lilly, and Roche. He has not received honoraria, travel reimbursements consulting fees or other personal payments.
